# Pathomimetic avatars reveal divergent roles of microenvironment in invasive transition of ductal carcinoma in situ

**DOI:** 10.1186/s13058-017-0847-0

**Published:** 2017-05-15

**Authors:** Mansoureh Sameni, Dora Cavallo-Medved, Omar E. Franco, Anita Chalasani, Kyungmin Ji, Neha Aggarwal, Arulselvi Anbalagan, Xuequn Chen, Raymond R. Mattingly, Simon W. Hayward, Bonnie F. Sloane

**Affiliations:** 1 Department of Pharmacology, Wayne State University School of Medicine, Detroit, MI 48201 USA; 2 Department of Biological Sciences, University of Windsor, Windsor, ON N9B 3P4 Canada; 3 Department of Surgery, NorthShore University HealthSystem Research Institute, Evanston, IL 60201 USA; 4 Department of Urologic Surgery, Vanderbilt University Medical Center, Nashville, TN 37232 USA; 5 Department of Physiology, Wayne State University School of Medicine, Detroit, MI 48201 USA; 6 Department of Oncology, Wayne State University School of Medicine, Detroit, MI 48201 USA; 7 Department of Cancer Biology, Vanderbilt University Medical Center, Nashville, TN 37232 USA

**Keywords:** Ductal carcinoma in situ, Myoepithelial cells, Fibroblasts, Tumor microenvironment, Proteolysis, 3D pathomimetic model, Heterotypic xenografts, Urokinase plasminogen activator, Interleukin 6

## Abstract

**Background:**

The breast tumor microenvironment regulates progression of ductal carcinoma in situ (DCIS) to invasive ductal carcinoma (IDC). However, it is unclear how interactions between breast epithelial and stromal cells can drive this progression and whether there are reliable microenvironmental biomarkers to predict transition of DCIS to IDC.

**Methods:**

We used xenograft mouse models and a 3D pathomimetic model termed *mammary architecture and microenvironment engineering* (MAME) to study the interplay between human breast myoepithelial cells (MEPs) and cancer-associated fibroblasts (CAFs) on DCIS progression.

**Results:**

Our results show that MEPs suppress tumor formation by DCIS cells in vivo even in the presence of CAFs. In the in vitro MAME model, MEPs reduce the size of 3D DCIS structures and their degradation of extracellular matrix. We further show that the tumor-suppressive effects of MEPs on DCIS are linked to inhibition of urokinase plasminogen activator (uPA)/urokinase plasminogen activator receptor (uPAR)-mediated proteolysis by plasminogen activator inhibitor 1 (PAI-1) and that they can lessen the tumor-promoting effects of CAFs by attenuating interleukin 6 (IL-6) signaling pathways.

**Conclusions:**

Our studies using MAME are, to our knowledge, the first to demonstrate a divergent interplay between MEPs and CAFs within the DCIS tumor microenvironment. We show that the tumor-suppressive actions of MEPs are mediated by PAI-1, uPA and its receptor, uPAR, and are sustained even in the presence of the CAFs, which themselves enhance DCIS tumorigenesis via IL-6 signaling. Identifying tumor microenvironmental regulators of DCIS progression will be critical for defining a robust and predictive molecular signature for clinical use.

**Electronic supplementary material:**

The online version of this article (doi:10.1186/s13058-017-0847-0) contains supplementary material, which is available to authorized users.

## Background

The breast cancer microenvironment is composed of epithelial tumor cells admixed with myoepithelial cells (MEPs); stromal cells, including fibroblasts as well as vascular and immune cells; and extracellular matrix (ECM) molecules [1–3]. Cross talk among breast epithelial and stromal cells, along with changes in both their gene expression and enzymatic activities, has been shown to be a driver of disease progression [4–6]. Hence, efforts are focused on targeting tumor-promoting factors of the breast microenvironment as an alternative therapeutic approach against metastatic and treatment-resistant breast cancers [7–9].

In normal breast tissue, MEPs support and promote differentiation and polarity of epithelial acinar structures and aid in expelling milk from acini into adjoining epithelial ducts [2, 10]. MEPs produce the basement membrane that separates the luminal epithelial cells from the surrounding ECM and exhibit a tumor-suppressive phenotype by inhibiting cell growth, invasion, and angiogenesis [10, 11]. Loss of the protective role of MEPs in the breast tumor microenvironment is correlated with the transition of ductal carcinoma in situ (DCIS) to invasive ductal carcinoma (IDC), a key event in the progression of breast cancer [12].

Cancer-associated fibroblasts (CAFs) have been shown to play a significant role in promoting breast cancer progression and metastasis through paracrine signaling [13–15]. CAFs secrete high levels of growth factors, chemokines, cytokines, and proteases that enhance tumor cell proliferation, angiogenesis, and invasion [16, 17]. In both in vivo and in vitro studies of DCIS, CAFs have been shown to induce an invasive DCIS phenotype in parallel with an increase in matrix metalloprotease (MMP)-14 expression and MMP-9 activity [18, 19]. We have observed that DCIS cell proliferation, ECM proteolysis, migration, and invasion are increased in the presence of normal fibroblasts induced to secrete hepatocyte growth factor (HGF) [20] and CAFs secreting interleukin 6 (IL-6) [21]. Others have also demonstrated a tumor-promoting role in breast and ovarian cancers for IL-6 secreted from CAFs [22, 23]. Both direct and indirect correlations between expression of IL-6 and upregulation of proteases involved in ECM degradation (e.g., cathepsin B, MMPs, and urokinase plasminogen activator [uPA]) and invasion have been reported [21, 24–27]. We have recently shown that paracrine IL-6 signaling between DCIS cells and CAFs facilitates tumor cell growth and migration in part through cathepsin B-mediated ECM degradation [21].

One of the challenges in treating patients with DCIS is the lack of reliable biomarkers that can predict transition of DCIS to IDC. In vivo mouse models, such as subcutaneous and orthotopic approaches including intraductal injection, have provided valuable tools to study DCIS progression [19, 28, 29]. It is often difficult, however, to delineate the complex molecular mechanisms that drive the invasive transition of DCIS using only in vivo models. In this respect, the advantage of heterotypic 3D in vitro models is that they can be designed to examine the cross talk between epithelial and stromal cells within the context of a defined microenvironment [30, 31]. Moreover, 3D in vitro models can be used to elucidate the role of noncellular microenvironmental factors (e.g., ECM molecules, pH, oxygen, biomechanical forces) on disease progression [20, 32–37]. We have used a 3D in vitro system for the study over time (4D) of breast cancer in the context of its microenvironment termed the *mammary architecture and microenvironment engineering (MAME) model* [30]. Coculture of various cell types in these pathomimetic avatars allows for recapitulation of in vivo architecture of breast cancer tissue and serves as a tractable platform to study and image cell-cell and cell-matrix interactions in real time (4D).

In the present study, we used both MAME and xenograft (orthotopic and subrenal capsule) models to examine the effects of MEPs and CAFs in regulating the invasive transition of DCIS cells. Our data demonstrate that the tumor-promoting effects of CAFs in vivo can be diminished by the presence of MEPs. Using MAME models, we further show that MEPs reduce the dysplastic phenotype of DCIS cells and inhibit CAF-induced ECM proteolysis and invasion by DCIS structures in vitro. Our MAME data also suggest that MEPs suppress the invasive transition of DCIS via increased plasminogen activator inhibitor 1 (PAI-1) secretion. Moreover, the effects of MEPs can supersede tumor-promoting CAFs by blocking IL-6 signaling pathways.

## Methods

### Materials

Reconstituted basement membrane (rBM; Cultrex reduced growth factor) was purchased from Trevigen (Gaithersburg, MD, USA). Dye-quenched collagen IV (DQ-collagen IV), DQ-collagen I, Alexa Fluor 546 phalloidin, Hoechst 33342, SlowFade reagent, polyclonal anti-p63 antibody, polyclonal cytokeratin 14 (CK14) antibody, fluorescein isothiocyanate-conjugated, affinity-purified donkey antimouse immunoglobulin G (IgG) and normal donkey serum, normal goat serum, the LIVE/DEAD® Viability/Cytotoxicity Kit, and SYBR® Green I were purchased from Thermo Fisher Scientific (Waltham, MA, USA). Lenti-RFP (red fluorescent protein [RFP]) and lenti-YFP (yellow fluorescent protein [YFP]) were purchased from Lentigen (Gaithersburg, MD, USA). PAI-1 protein was obtained from EMD Chemicals (Gibbstown, NJ, USA). Antihuman laminin-5 γ^2^-chain, domain III (EMD Millipore, Billerica, MA, USA) recognizes laminin-332 (*laminin-5* in previous nomenclature) and its isoforms laminin-3A32 (laminin-5A) and laminin-3B32 (laminin-5B). Recombinant plasminogen activator inhibitor 1 (rPAI-1) protein, mutated to increase its stability, was purchased from EMD Millipore (Billerica, MA, USA). Polyclonal anti-uPA and polyclonal anti-urokinase plasminogen activator receptor (anti-uPAR) antibodies were kind gifts from Dr. Gunilla Hoyer-Hanson (Finsen Centre, Copenhagen, Denmark). Monoclonal antibodies to uPAR (ATN-617) were kindly provided by Dr. Andrew Mazar (Northwestern University, Evanston, IL, USA). Human cytokine antibody arrays (AAH-CYT-G5) were obtained from RayBiotech (Norcross, GA, USA). Human affinity-purified IL-6 neutralizing antibody (nAb; AF-206-NA) was purchased from R&D Systems (Minneapolis, MN, USA), and anti-glyceraldehyde 3-phosphate dehydrogenase (anti-GAPDH) was obtained from EMD Millipore (Billerica, MA, USA). Monoclonal anti-CD10 antibody was purchased from Abcam (Cambridge, MA, USA). Acrylamide, nitrocellulose membranes, and protein assay reagents were obtained from Bio-Rad Laboratories (Hercules, CA, USA). Prestained protein markers and chemiluminescence immunoblotting detection kits were purchased from PerkinElmer (Boston, MA, USA). HRP-labeled goat antirabbit and goat antimouse IgG were obtained from Pierce Biotechnology (Rockford, IL, USA). Mammary epithelial basal medium (MEBM) without phenol red and mammary epithelial growth medium (MEGM) SingleQuots were purchased from Lonza (Basel, Switzerland). HyClone FBS was obtained from GE Healthcare Life Sciences (Logan, UT, USA). CB17/Icr/Hsd^*scid*^ severe combined immunodeficiency (SCID) mice were purchased from Harlan Laboratories (Indianapolis, IN, USA). Masson’s Trichrome Stain Kit, biotinylated streptavidin-HRP secondary antibody, and 3,3′-diaminobenzidine tetrahydrochloride were obtained from Dako (Carpinteria, CA, USA). Human breast tissue microarray (BR8011) from US Biomax (Rockville, MD, USA). ImmPRESS™ antimouse IgG, normal horse serum, ImmPACT™ NovaRED™ substrate, avidin-biotin complex-HRP complex, and VectaMount mounting medium were obtained from Vector Laboratories (Burlingame, CA, USA). Sequencing grade modified trypsin was purchased from Promega (Madison, WI, USA). The Dionex μ-precolumn C18 reversed-phase cartridge and Acclain PepMap100 C18 reversed-phase analytical column were purchased from Thermo Fisher Scientific (Sunnyvale, CA, USA). High-performance liquid chromatography (HPLC) grade water and acetonitrile (Optima) were purchased from Fisher Scientific (Pittsburgh, PA, USA). Bradford protein assay kits were obtained from Thermo Fisher Scientific. Bovine serum albumin, antibiotics, monoclonal anti-α-smooth muscle actin (anti-αSMA), Triton X-100, protease inhibitor cocktail, and all other chemicals, unless otherwise stated, were purchased from Sigma-Aldrich (St. Louis, MO, USA). Antihuman CK17 antibody, clone E3, was purchased from Dako.

### Cells and cell culture

MCF-10A human breast cell variants [38] and WS-12T human breast fibroblasts [39] were originally established at and obtained from the Michigan Cancer Foundation (the institutional precursor to the Barbara Ann Karmanos Cancer Institute, Detroit, MI, USA). SUM102 human breast DCIS cells [40] and N1ME human breast MEPs were kind gifts of Dr. S. Ethier (Medical University of South Carolina, Charleston, SC, USA) and Dr. K. Polyak (Dana-Farber Cancer Institute, Boston, MA, USA), respectively. MCF-10A human breast epithelial cells were maintained in DMEM/F-12 supplemented with 5% horse serum, 100 μg/ml insulin, and 5 ng/ml epidermal growth factor [41]. MCF10.DCIS and SUM102 human breast DCIS cells were maintained in DMEM/F-12 supplemented with 5% horse serum and in Ham’s F-12 media supplemented with 10% FBS, respectively [20]. N1ME MEPs, transduced with murine stem cell virus-puro-human telomerase reverse transcriptase and selected under 0.4 μg/ml puromycin, were maintained at low passage in MEGM without phenol red [19]. WS-12T CAFs were maintained in DMEM-F-12 with 10% FBS [42]. For 3D MAME cultures, MEBM-phenol red free supplemented with MEGM was used. MCF10.DCIS and SUM102 were transduced with lenti-RFP, and WS-12T was transduced with lenti-YFP, to distinguish cell types in 3D cocultures. Cell lines were authenticated using the STR PowerPlex 16 System (Promega) and routinely screened for mycoplasma by microscopy (MycoFluor; Thermo Fisher Scientific) and reverse transcription-polymerase chain reaction (LookOut; Sigma-Aldrich). Further characterization of N1ME cells was performed by immunostaining and immunoblotting for basal markers αSMA, p63, CK14, CK17, and CD10 (Additional file 1: Figure S1).

### Tissue recombinant xenografts

In vivo experiments were performed with the approval of the relevant institutional animal care and use committee. All procedures were reviewed and approved and conform to all local, state, and U.S. federal regulatory standards. CB17/Icr/Hsd^*scid*^ mice were used for xenograft studies. MCF10.DCIS (2.0 × 10^5^), N1ME (1.0 × 10^5^), and WS-12T (4 × 10^4^) alone or in combination were mixed in type I collagen, and, after overnight incubation at 37 °C, they were grafted either under the renal capsule or orthotopically within the fourth inguinal mammary gland of eight intact female SCID mice [43, 44]. The use of two graft sites allows an internal assessment of consistency of outcomes. The renal capsule site was chosen because of its high level of vascularity and associated graft take rate, whereas the orthotopic site has greater biological relevance. Internally consistent observations at both sites strengthen confidence in the data generated. Surviving host mice (*n* = 6–8) were killed after 8 weeks. The kidneys were removed, and grafts were cut into halves and photographed before being processed for histology (hematoxylin and eosin stain, Masson’s trichrome stain for collagen, and laminin-332) and immunofluorescence (αSMA, p63, and CK14). Tumors in the mammary gland were processed in the same manner as the kidney grafts. Graft dimensions were measured, and the tumor volume was calculated using the formula volume = width × length × depth × π/6.

### Preparation and imaging of live 3D MAME cultures

A seeding ratio of 5:2.5:1 of DCIS cells, MEPs, and CAFs, respectively, was used for 3D MAME cultures. Briefly, cells were seeded onto glass coverslips coated with 50 μl of rBM, overlaid with 2% rBM, and grown for periods ranging from 4 to 21 days. Optical sections through the entire depth of the 3D structures were acquired using a Zeiss LSM 510 Meta nonlinear optical (NLO) confocal microscope (Carl Zeiss Microscopy, Thornwood, NY, USA) with a water immersion objective and reconstructed in 3D using Volocity software (PerkinElmer). Volumes of 3D MAME structures were quantified using Volocity software. Where indicated, 3D MAME cultures were modified as follows: (1) CAFs were embedded in a layer of collagen I containing DQ-collagen I directly underneath the rBM layer, or (2) CAFs were embedded within the rBM layer containing DQ-collagen IV [30].

### Immunostaining of 3D MAME cultures

Cells were grown in 3D on rBM-coated coverslips, permeabilized, and stained for human laminin-332 and actin according to our previously published procedures [41]. Antibodies against human laminin-332, not mouse, were used to determine expression of the human protein from cells grown in rBM of mouse origin. Cells were incubated with mouse antihuman laminin-332 (20 μg/ml) or preimmune mouse IgG (110 μg/ml) overnight at 4 °C. After being washed with PBS, the cells were incubated with a 1:1000 dilution of Alexa Fluor-conjugated, affinity-purified donkey antimouse IgG containing 5% normal donkey serum and phalloidin (1:50; actin staining) for 1 h.

### Live-cell proteolysis assay

3D MAME cultures were prepared and imaged as described above with the addition of 25 μg/ml DQ-collagen IV to the 50 μl of rBM [30]. Prior to imaging, nuclei were stained with Hoechst 33342. Optical sections in 16 contiguous fields through the entire depth of the 3D structures were acquired on a Zeiss LSM 510 Meta NLO confocal microscope using a water immersion objective. 3D reconstructions of optical sections of each structure were generated using Volocity software. Fluorescent intensities of degraded dye-quenched collagen IV fragments (dDQ-IV, green) are from the entire structure; that is, they are the sum of intensities in individual optical sections quantified. Data are represented as total intensity of dDQ-IV/cell (based on number of nuclei), as total volume of dDQ-IV (μm^3^), or as total volume of dDQ-IV (μm^3^ × 10^3^)/cell.

### Treatment of 3D MAME cultures with conditioned media

Conditioned media were collected from MEPs grown in 2D monolayer cultures for 3 days and centrifuged at 150 × *g* to remove cell debris. MCF10.DCIS and SUM102 cells were then grown in 3D MAME cultures with the addition of 25 μg/ml DQ-collagen IV (as described above) for 16 days in the presence and absence of MEP-conditioned media mixed at a ratio of 1:2 with fresh MEGM. Conditioned media were added to 3D MAME cultures prior to the addition of a 2% rBM overlay and replaced every other day. On days 8 and 16, live-cell imaging and quantification of 3D MAME volume were performed as described above.

### Immunoblots of 3D MAME cultures

3D cultures of MEPs, 3D cultures of MCF10.DCIS, and 3D cocultures of DCIS-MEP were grown in 60-mm dishes for 8 days as described above. Conditioned media were collected at day 8, centrifuged at 150 × *g* to remove cell debris, and concentrated through Ultrafree 100 K and then 3 K concentrators (EMD Millipore). Cell lysates were prepared according to our previously published procedures [20]. Cell lysates and conditioned media were loaded on the basis of DNA concentrations of the cell lysates [45]. Samples were separated by SDS-PAGE; transferred onto nitrocellulose membranes; and immunoblotted for uPA, PAI-1, and GAPDH (loading control) [46].

### Treatment of 3D MAME cultures with rPAI-1 and ATN-617

Live-cell proteolysis assays were performed on MCF10.DCIS or MCF10.DCIS-lenti-RFP cells grown in 3D MAME cultures in the absence (control) or presence of rPAI-1 (250 nM), preimmune IgG (10 μg/ml), or the uPAR-blocking monoclonal antibody ATN-617 (10 μg/ml), all added at the time of seeding [47]. Culture medium was replaced every other day with fresh medium containing 2% rBM and either rPAI-1, preimmune IgG, or ATN-617. Live-cell imaging and quantification of 3D MAME volume and DQ degradation were performed as described above.

### Treatment of 3D MAME cultures with IL-6 nAb

MCF10.DCIS lenti-RFP or SUM102 cells were grown with CAFs in 3D MAME cultures as described above in the presence of 100 ng/ml IL-6 nAb [21]. Negative controls were run with an equivalent concentration of isotype control. Culture medium was replaced every other day with fresh medium containing 2% rBM and 100 ng/ml IL-6 nAb or an equivalent concentration of isotype control and then imaged on days 2, 4, 6, and 8. Optical sections through the entire depth of the 3D structures were acquired on a Zeiss LSM 510 Meta NLO confocal microscope using a water immersion objective. Volocity software was used to generate 3D reconstructions and quantify the volume of 3D MAME structures.

### Tissue microarray

A tissue microarray that included samples of human breast DCIS and adjacent normal breast (BR8011; US Biomax) was stained for uPAR and laminin-332. The microarray was deparaffinized; hydrated; and then incubated with 7 μg/ml ATN-617 or 10 μg/ml laminin-332 antibodies overnight at 4 °C, washed for 5 minutes, and incubated with ImmPRESS reagent for 30 minutes before being washed with PBS. The tissue microarray was incubated with ImmPACT™ NovaRED substrate for 10 minutes, washed, counterstained with hematoxylin, and mounted in nonaqueous mounting medium (VectaMount).

### Proteomic analyses

2D and 3D cultures of MEPs, 3D cultures of MCF10.DCIS, and 3D cocultures of DCIS-MEP were grown in 60-mm dishes for 8 days. Conditioned media were collected at day 8, centrifuged at 150 × *g* to remove cell debris, and concentrated through Ultrafree 100 K and then 3 K concentrators. The same volume of growth media from uncoated and rBM-coated 60-mm dishes was also concentrated and used as a control. Protein concentration of conditioned media from 2D and 3D cultures of MEPs, 3D cultures of MCF10.DCIS, and 3D cocultures of DCIS-MEP and control media was measured using Bradford reagent according to the manufacturer’s procedure. Sample proteins were precipitated with cold acetone. The washed pellets were then resuspended in 50 mM trimethylammonium bicarbonate, reduced, alkylated, and digested with sequencing-grade trypsin (at an estimated 1:20 wt/wt ratio) as previously described [48]. The resulting peptides were analyzed by nanoscale liquid chromatography coupled to tandem mass spectrometry. Peptides were separated on a reversed-phase C18 column using the Dionex Ultimate™ HPLC system and a QSTAR XL mass analyzer (Applied Biosystems, Foster City, CA, USA) as described elsewhere [48]. Mass spectrometry was performed from *m/z* 400 to 1500 for 1 second, followed by product ion scanning on the two most intense multiply charged ions. The peak lists were submitted to the Mascot server (Matrix Science, Boston, MA, USA) to search against the UniProt database for *Homo sapiens* with carbamidomethyl as a fixed modification and oxidation and *N*-acetylation (protein amino-terminus) as variable modifications, 0 or 1 missed tryptic cleavage, 100-ppm mass tolerance for precursor ions, and 0.6 Da for the fragment ions.

### Cytokine antibody array

DCIS cells, MEPs, and CAFs were grown either alone or in combination in 3D MAME cultures on 60-mm dishes for 8 days. Media from days 4 and 8 were pooled, centrifuged at 150 × *g* to remove cell debris, and analyzed for cytokine secretion using antibody arrays as specified by the manufacturer. Growth media not conditioned by MAME cultures were used as a negative control.

### Statistics

Statistical analyses were carried out using Prism version 7.0 software (GraphPad Software, La Jolla, CA, USA). One-way analysis of variance (ANOVA) and two-way ANOVA were used, where specified, to compare means between the groups, followed by Bonferroni post hoc analysis to correct for multiple comparisons. Other statistical analyses were done by two-sided Student’s *t* test. For all studies, *p* ≤ 0.05 was considered statistically significant.

A sample size of three mice per group was used. Each mouse contributed to more than one observation, namely two sites, which provided enough power (approximately 80%) to determine differences between groups. The statistical method used was ANOVA. Animal experiments were not randomized, but were blinded to the principal investigator.

## Results

### MEPs reduce the volume and malignant phenotype of DCIS xenografts in vivo in two preclinical mouse models: mammary fat pad and renal capsule

**Fig. 1:**
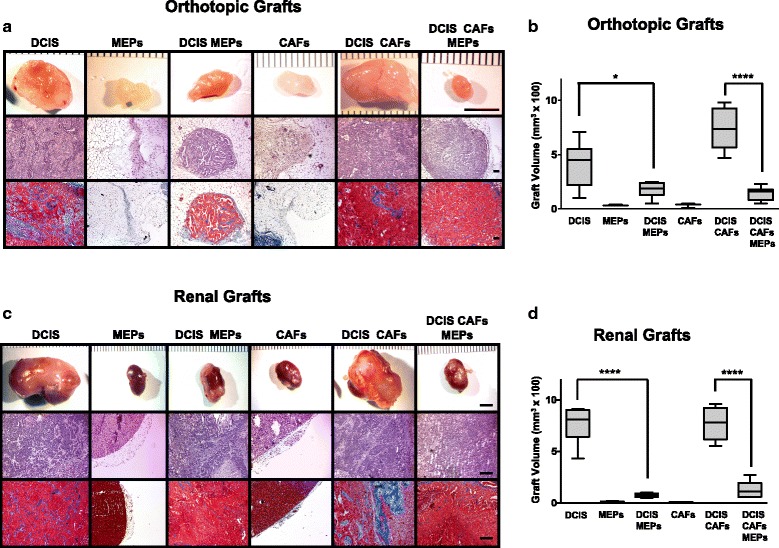
Myoepithelial cells (MEPs) reduce volume and malignant phenotype of ductal carcinoma in situ (DCIS) xenografts. MCF10.DCIS (DCIS) cells, N1ME cells (MEPs), and/or WS-12T (cancer-associated fibroblasts [CAFs]) were implanted under the renal capsule or orthotopically within the mammary fat pad of female severe combined immunodeficiency mice and evaluated after 8 weeks. **a** Representative orthotopic xenografts. Scale bar = 5 mm (*top row*); hematoxylin and eosin (H&E) staining; scale bar = 100 μm (*middle row*); and Masson’s trichrome staining for collagen (*blue*); scale bar = 100 μm (*bottom row*). **b** Volume of orthotopic xenografts (*n* = 3–8). **c** Representative renal xenografts. Scale bar = 5 mm (*top row*); H&E staining; scale bar = 100 μm (*middle row*); and Masson’s trichrome staining for collagen (*blue*); scale bar = 100 μm (*bottom row*). **d** Volume of renal xenografts (*n* = 6, except for CAFs alone, which were *n* = 2 and not used in statistical analyses). Data are presented as box-and-whisker plots where the box represents the interquartile range and whiskers represent minimum and maximum values. * *p* ≤ 0.05; **** *p* ≤ 0.0001 as determined by one-way analysis of variance and Bonferroni’s multiple comparisons test. Immunostaining and immunoblotting results for basal markers are shown in Additional file 1: Figure S1

We investigated the suppressive role of MEPs on DCIS in the absence and presence of tumor-promoting CAFs using two complementary human tumor xenograft models. DCIS cells, MEPs, and CAFs were grafted in various combinations in intact female SCID mice: (1) orthotopically within the fourth inguinal mammary gland or (2) under the renal capsule. Tissue recombination and grafting are well-established approaches to explore both development and disease and have been used to study a range of organs, including the mammary gland [43, 49]. Grafting can be performed either orthotopically, providing the most relevant context, or at the subrenal capsule site, providing better vascularization and take rate. Our findings were consistent between graft sites, although grafts in the highly vascularized renal capsule tended to be larger, likely reflecting a higher initial take rate for cells. Coimplantation of normal MEPs with DCIS cells resulted in orthotopic (Fig. 1a, *top row*, and b) and renal capsule xenografts (Fig. 1c, *top row*, and d) that were significantly smaller in size than xenografts of DCIS cells alone. Coimplantation of DCIS cells with CAFs significantly increased volumes of orthotopic xenografts (Fig. 1a, *top row*) (*p* < 0.001); however, changes in histology, such as tissue disorganization, that are consistent with a more malignant phenotype were observed in both orthotopic and renal capsule xenografts (Fig. 1a and c, *middle rows*). To verify the presence of MEPs in the xenografts, we stained for and show colocalization of three basal markers (i.e., αSMA, p63, and CK14) (Additional file 1: Figure S1). To investigate the effects of MEPs on the malignant phenotype of DCIS tumors in the presence of CAFs, all three cell types were coimplanted in both xenograft models. Even in the presence of CAFs, MEPs significantly reduced the tumor sizes to levels similar to xenografts of DCIS and MEPs without CAFs (Fig. 1b and d). MEPs also reduced collagen deposition in the stroma of DCIS xenografts (Fig. 1a and c, *bottom rows*). Because fibroblasts are involved in collagen deposition during the remodeling of the ECM in breast cancer progression and invasion [50], these data further demonstrate the overriding effects of MEPs on CAFs to suppress the malignant progression of DCIS.

### MEPs reduce the size and dysplastic phenotype of DCIS structures grown in 3D MAME cultures

**Fig. 2:**
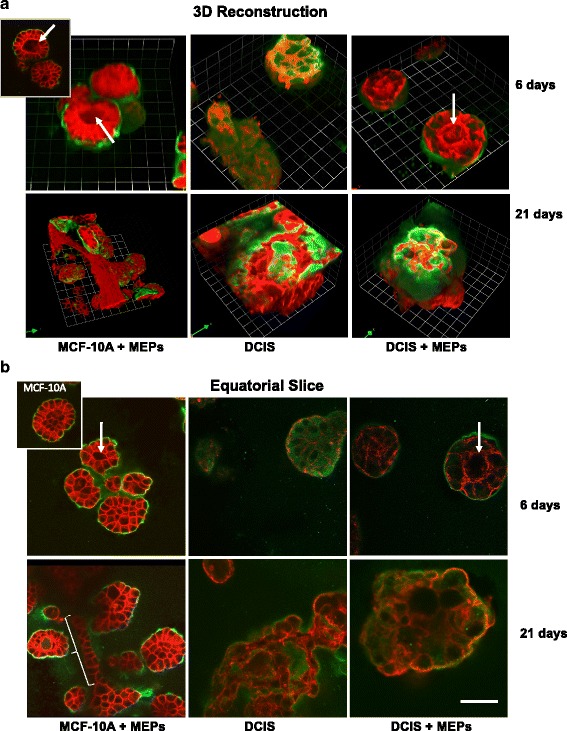
Myoepithelial cells (MEPs) in mammary architecture and microenvironment engineering (MAME) cocultures facilitate organization of MCF-10A acinar structures and reduce size and dysplastic phenotype of ductal carcinoma in situ (DCIS) structures. Nontransformed breast epithelial cells (MCF-10A) and MCF10.DCIS cells (DCIS) were grown in MAME cultures in the absence and presence of N1ME cells (MEPs) and imaged live at 6 and 21 days. *Red* and *green* represent phalloidin staining of the actin cytoskeleton and immunostaining for human laminin-332, respectively. **a** 3D reconstructions of Z-stack images captured at 6 (*top rows*) and 21 (*bottom rows*) days in MAME culture and generated with Volocity software. One grid unit = 23 μm. *Inset* in *top row*, *left column*, is a representative en face view of MCF-10A plus MEPs. **b** Confocal slices at the equatorial plane of the structures captured at 6 (*top rows*) and 21 (*bottom rows*) days in MAME cultures. Scale bar = 50 μm. *Inset* in *top row*, *left column*, is a representative image of MCF-10A cells grown alone in MAME culture at day 6. Images are representative of at least three independent experiments. Acinar lumens (*white arrows*) and tubular morphology (*white bracket*) are indicated. Immunostaining for laminin-332 and videos of MCF-10A 3D acinar structures are shown in Additional file 1: Figure S1, Additional file 2: Video S1, Additional file 3: Video S2, and Additional file 4: Figure S2



Structural integrity and function of the mammary gland are dependent on interactions between the breast epithelium, basement membrane, and surrounding ECM [3]. MAME 3D pathomimetic models are designed to mimic the in vivo architecture of breast tissue under controlled in vitro conditions [30]. We employed MAME cultures to identify changes in the morphology of breast epithelial cells in the presence and absence of MEPs. One of the markers we examined was laminin-332 (*laminin-5* in previous nomenclature), which plays crucial roles in cell adhesion, migration, and differentiation [51]. Although other laminins are found in the basement membrane, laminin-332 is of interest because it is expressed in the invading cells of various malignancies, including breast ductal carcinomas and their associated MEPs, and is coexpressed with uPAR in colorectal cancer cells [52]. Furthermore, downregulation of laminin-332 in bladder carcinoma cells increases the number of invadopodia and ECM degradation, suggesting a role for laminin-332 in negatively regulating proteolysis and cell invasion [53]. We also show the presence of laminin-332 in our DCIS xenografts, with increased staining observed in the presence of MEPs (Additional file 1: Figure S1). In 6-day MAME cocultures of nontransformed breast epithelial cells (MCF-10A) and MEPs, well-organized acinar structures enclosing a central lumen were observed, with laminin-332 localized around the acinar structures at the epithelial-stromal interface (Fig. 2a and b, *top row*, *left column*). These observations can be more clearly visualized in videos of the 3D structures (Additional file 2: Video S1). The MCF-10A acinar structures are similar to those grown in 3D in the absence of MEPs (Fig. 2b, *top row*, *left column inset*) [30, 41]. By day 21, tubelike structures were observed linking the 3D acinar structures (Fig. 2a and b, *bottom row*, *left column*), resembling the architecture of normal breast tissue. The tubular morphology can be seen more clearly when the 3D reconstructions are rotated (Additional file 3: Video S2). By comparison, the acinar structures formed in 6- and 21-day MAME cultures of DCIS cells alone were larger, lacked lumens, and were less organized (as shown by actin staining), resembling a dysplastic phenotype (Fig. 2a and b, *middle column*). Although human DCIS cells form 3D structures that are functional in that they produce and secrete human laminin when grown in murine rBM, the pattern of laminin-332 staining was diffuse and localized within, rather than at the edge of, the acinar structures. Diffuse staining for laminin-332 in DCIS was also observed in a tissue microarray (Additional file 4: Figure S2). In the presence of MEPs, 3D DCIS structures were smaller and more organized, even in some cases having a lumen (Fig. 2a and b, *right column*). Moreover, the diffuse staining of laminin-332 was reduced and localized at the periphery of the acinar structures. These data suggest that MEPs play a role in the organization of DCIS cells and in reverting their dysplastic phenotype.

**Additional file 2: Video S1.** MEPs facilitate organization of acinar structures by MCF-10A cells in 6-day cocultures. 3D reconstruction of nontransformed breast epithelial cells (MCF-10A) grown in MAME cultures in the presence of N1ME cells (MEPs) and imaged live at day 6. *Red* and *green* represent phalloidin staining of the actin cytoskeleton and immunostaining for human laminin-332, respectively. One grid unit = 23 μm. (MOV 714 kb)




**Additional file 3: Video S2.** MEPs facilitate organization of ductal structures by MCF-10A cells in 21-day cocultures. 3D reconstruction of nontransformed breast epithelial cells (MCF-10A) grown in MAME cultures in the presence of N1ME cells (MEPs) and imaged live at day 21. *Red* and *green* represent phalloidin staining of the actin cytoskeleton and immunostaining for human laminin-332, respectively. One grid unit = 23 μm. (MOV 22738 kb)


### MEPs reduce invasiveness of DCIS structures grown in MAME cultures

**Fig. 3:**
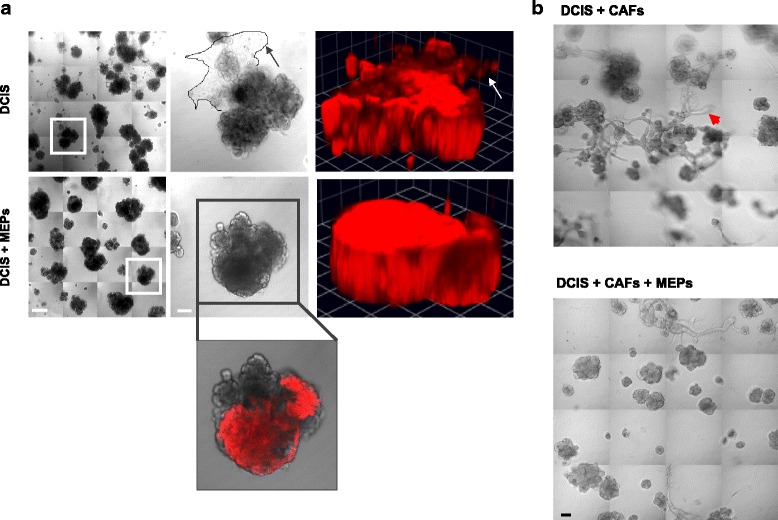
Myoepithelial cells (MEPs) reduce invasive phenotype of ductal carcinoma in situ (DCIS) structures formed in mammary architecture and microenvironment engineering (MAME) cultures. **a** MCF10.DCIS-lenti-RFP (DCIS) were seeded in MAME cultures alone or with N1ME cells (MEPs) and imaged live at day 16. *Left columns* are tiled images of 16 contiguous differential interference contrast (DIC) fields. Scale bar = 100 μm. *Middle columns* are magnified DIC images of the *boxed areas* in the *left columms* and illustrate invasive outgrowths from DCIS structures in the absence of MEPs (*outlined area* and *arrow* in *top row*, *middle column*). Scale bar = 380 μm. *Right columns* are 3D reconstructions of Z-stack images of DCIS structures (*red*). One grid unit = 90 μm (*arrow* in *top row*, *right column*, corresponds to the same invasive outgrowth highlighted by *arrow* in *top row*, *middle column*). The *inset* in the *bottom row*, *middle column*, shows an overlay of unlabeled MEPs (DIC) and the absence of outgrowths in DCIS structures (*red*) when cocultured with MEPs. Scale bar = 100 μm. **b** MCF10.DCIS cells (DCIS) and WS-12T cells (cancer-associated fibroblasts [CAFs]) were seeded with or without N1ME cells (MEPs) in MAME cultures and imaged live at day 8. *Arrowhead* points to an invasive outgrowth. Images are 16 contiguous tiled fields (scale bar = 90 μm). Images are representative of at least three independent experiments. Views of 3D reconstructions from a number of angles are shown in Additional file 5: Figure S3

In normal breast tissue, MEPs contribute to the structural organization of the breast tissue and prevent epithelial cells from migrating and invading into the ECM [10]. We investigated whether MEPs could reverse the invasive phenotype of DCIS cells over a 16-day period of growth in MAME cultures. Owing to the large size of the DCIS structures, we captured differential interference contrast (DIC) images at low magnification from 16 contiguous fields of view and assembled them into a montage (Fig. 3a, *left columns*, and b). By using montages, we can concomitantly compare multiple DCIS structures. DCIS cells cultured alone formed irregularly shaped 3D structures and exhibited extensive invasive outgrowths (Fig. 3a, *top row*). At higher magnification, outgrowths could be seen to be comprised of a sheet of cells that protruded from DCIS structures into the surrounding ECM (Fig. 3a, *outlined area* and *arrow* in the *top row*, *middle column*). In 3D reconstructed images, invasive outgrowths projecting from DCIS structures into the ECM resulted in the surface of DCIS structures appearing irregular (Fig. 3a, *top row*, *right column*; *arrow* in *top row*, *right column*, corresponds to *arrow* in *top row*, *middle column*). These projecting 3D multicellular outgrowths (*arrows*) are further illustrated in images of 3D reconstructions shown from various angles (Additional file 5: Figure S3). In the presence of MEPs, DCIS structures had fewer invasive outgrowths (Fig. 3a, *bottom row*), as is more apparent at a higher magnification (Fig. 3a, *bottom row*, *middle column*). In 3D reconstructed images (Fig. 3a, *bottom row*, *right column*), the cohesiveness of the structures is evident by the smooth surface (Fig. 3a, *bottom row*, *right column*, and Additional file 5: Figure S3). In DIC images, unstained MEPs were seen surrounding the DCIS cells in the cocultures (Fig. 3a, *inset* in *bottom row*, *middle column*), consistent with MEPs attenuating the invasive potential of the DCIS cells. In the presence of tumor-promoting CAFs (ratio of DCIS cells to CAFs was 5:1), there were numerous invasive outgrowths formed between DCIS structures (Fig. 3b, *top row*, *red arrowhead*); these CAF-induced outgrowths were suppressed in the presence of MEPs (Fig. 3b, *bottom row*).

### MEPs reduce ECM proteolysis by DCIS structures grown in MAME cultures

**Fig. 4:**
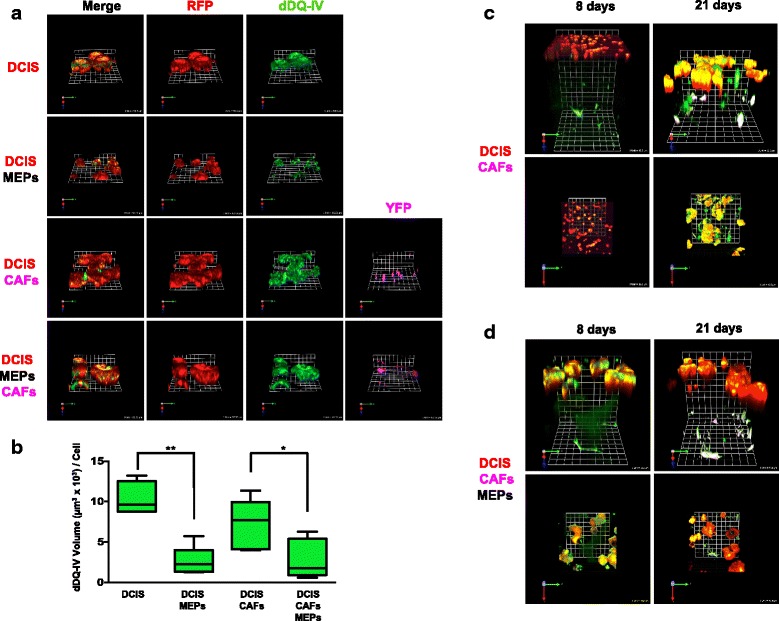
Myoepithelial cells (MEPs) reduce proteolysis of extracellular matrix (ECM) and invasive phenotype of ductal carcinoma in situ (DCIS) structures formed in mammary architecture and microenvironment engineering (MAME) and modified MAME cultures, respectively. **a** Representative angled en face views of 3D reconstructions of structures formed by MCF10.DCIS-lenti-RFP (DCIS, *red*) cells seeded alone (*top row*) or in coculture with N1ME (MEPs, unlabeled; *second row*), WS-12T-lenti-YFP (cancer-associated fibroblasts [CAFs], *pseudocolored fuchsia*; *third row*), or both CAFs and MEPs (*bottom row*). The reconstituted basement membrane (rBM) overlay cultures contained dye-quenched collagen IV (DQ-collagen IV) and were imaged live at 21 days. One grid unit = 92 μm. **b** Volume of degraded dye-quenched collagen IV (dDQ-IV, *green*) per cell in the 4 MAME culture variants was quantified in 64–96 fields (16 contiguous fields per experiment from 4–6 independent experiments) with Volocity software. Unlabeled cells were used to eliminate possible interference with analysis of fluorescent dDQ-IV degradation products. Degradation products were quantified per cell number as determined by counting nuclei labeled with Hoechst 33342 in 3D reconstructions assembled from optical sections through the entire depth of the cultures. Intensities of dDQ-collagen IV fragments in 3D are the sum of intensities in individual optical sections. Using the nuclei counts, these data were then calculated as volume per cell [(μm^3^ × 10^3^)/cell]. Data are presented as box-and-whisker plots where the box represents the interquartile range and whiskers represent minimum and maximum values. * *p* ≤ 0.05; ** *p* ≤ 0.01 as determined by one-way analysis of variance and Bonferroni’s multiple comparisons test (*n* = 4–6). Representative angled (**c** and **d**, *top rows*) and en face (**c** and **d**, *bottom rows*) views of 3D reconstructions of 8- and 21-day MAME cultures with the following culture modifications: WS-12T-lenti-YFP (CAFs, *pseudocolored fuchsia*) were embedded in a bottom layer of collagen I containing DQ-collagen I (dDQ-colI, *green*) and MCF10.DCIS-lenti-RFP (DCIS, *red*) cells with (**c**; 1 grid unit = 92 μm) and without (**d**; 1 grid unit = 90 μm) MEPs were seeded onto a second layer composed of rBM containing DQ-collagen IV (dDQ-IV, *green*). An overlay of 2% rBM was placed on top of the cocultured cells. Images are representative of at least three independent experiments. Additional results are shown in Additional file 6: Figure S4

We and other investigators have shown that incorporation of fibroblasts into 3D rBM cultures can recapitulate effects of the tumor microenvironment on malignant progression of DCIS, including enhancing ECM proteolysis [20, 54]. In the present study, we examined whether MEPs would reduce ECM proteolysis by DCIS structures formed in MAME cultures in the absence and presence of CAFs. Some variation could be observed as early as 8 days in culture in the size of DCIS structures and their degree of DQ-collagen IV degradation when grown alone versus in combination with CAFs and MEPs (Additional file 6: Figure S4). Differences were more evident after 21 days (Fig. 4 and Additional file 6: Figure S4). DCIS cells grown alone formed large dysplastic structures that degraded DQ-collagen IV (green) and invaded into the surrounding matrix (Fig. 4a, *top row*). Addition of MEPs suppressed the invasive phenotype of DCIS structures and reduced degradation of DQ-collagen IV (Fig. 4a and Additional file 6: Figure S4, *second rows*). Conversely, addition of CAFs enhanced the dysplastic and invasive phenotypes and the size of DCIS structures (Fig. 4a and Additional file 6: Figure S4, *third rows*). Moreover, in MAME triple cocultures of DCIS, MEPs, and CAFs, MEPs maintained partial suppression of DCIS progression and invasiveness, despite the presence of the tumor-promoting CAFs (Fig. 4a and Additional file 6: Figure S4, *bottom rows*). We quantified the DQ-collagen IV degradation products in a minimum of 64 fields for each of the 4 different MAME cultures and confirmed that the MEPs significantly suppressed DQ-collagen IV proteolysis (i.e., volume of degradation products per cell) by DCIS in the absence and presence of CAFs (Fig. 4b). We also examined a modified layered MAME in which CAFs were embedded in a layer of collagen I plus DQ-collagen I directly underneath the rBM layer seeded with DCIS cells. The modified MAME model reflects the location of fibroblasts prior to their infiltration into the tumor and allows for the examination of cell migration and invasion through the ECM. The distance between DCIS cells and CAFs was reduced after 21 days of coculture, consistent with movement of these cells toward each other (Fig. 4c). After 21 days of coculture of DCIS cells, CAFs, and MEPs, the invasive phenotype, including degradation of DQ-collagen I and IV, was attenuated (Fig. 4d), further supporting a role for MEPs in suppressing the tumor-promoting effects of CAFs on DCIS progression.

### MEP-conditioned media reduce dysplastic phenotype of DCIS structures and ECM proteolysis

**Fig. 5:**
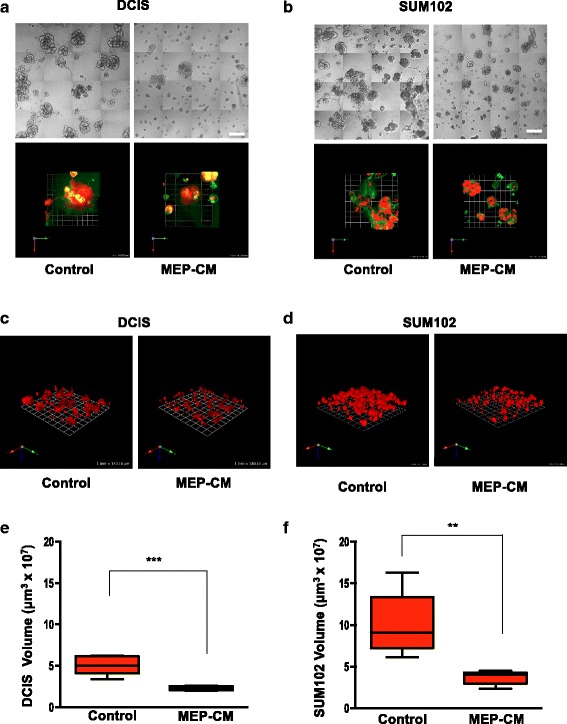
Myoepithelial cell-conditioned media (MEP-CM) reduce dysplastic phenotype of ductal carcinoma in situ (DCIS) structures formed in mammary architecture and microenvironment engineering (MAME) cultures. DCIS cells were seeded in reconstituted basement membrane overlay cultures containing dye-quenched collagen IV (DQ-collagen IV) in the absence (control) or presence of MEP-CM and imaged live at day 16. Differential interference contrast images are 16 contiguous tiled fields of MCF10.DCIS-lenti-RFP (DCIS; **a**, *top rows*) and SUM102-lenti-RFP (SUM102; **b**, *top rows*) structures. Scale bars = 180 μm. Fluorescence images are en face views of 3D reconstructions of DCIS (**a**, *bottom rows*) and SUM102 (**b**, *bottom rows*) structures (*red*) and their associated DQ-collagen IV degradation products (*green*). One grid unit = 45 μm. Representative angled views of 3D reconstructions illustrating volume of DCIS (**c**) and SUM102 (**d**) structures (*red*). One grid unit = 180 μm. Volumes of DCIS (**e**, *n* = 6) and SUM102 (**f**, *n* = 5) structures were quantified in 64 fields (16 contiguous fields per experiment from 4 independent experiments). Data are presented as box-and-whisker plots where the box represents the interquartile range and whiskers represent minimum and maximum values. ** *p* ≤ 0.007; *** *p* ≤ 0.0005 as determined by unpaired *t* test, two-sided. Images are representative of at least three independent experiments. Additional results are shown in Additional file 7: Figure S5, Additional file 8: Video S3, Additional file 9: Video S4, and Additional file 10: Figure S6



MEPs suppress invasion of tumor cells into the ECM by the secretion of anti-invasive factors [11, 55]. To determine whether factors secreted from the MEPs suppress the dysplastic phenotype of DCIS cells, we treated two different DCIS cell lines, MCF10.DCIS-lenti-RFP (DCIS) and SUM102-lenti-RFP (SUM102), with media conditioned by MEPs (MEP-CM). Like MCF10.DCIS cells, SUM102 cells form large dysplastic structures in 3D MAME cultures that resemble DCIS lesions seen in vivo [20] and degrade DQ-collagen IV [20, 30]. At day 16, as illustrated in the montage of 16 contiguous DIC images shown in Fig. 5a and b (*top rows*), MEP-CM reduced the size of DCIS and SUM102 structures as well as their invasive outgrowths and DQ-collagen IV degradation (*green*; Fig. 5a and b, *bottom rows*). The decrease in volume of both DCIS and SUM102 structures in the presence of MEP-CM is apparent in 3D reconstructed images captured at days 8 (Additional file 7: Figure S5) and 21 (Fig. 5c and d). Quantitative analyses confirmed a significant reduction in the volumes of both DCIS (Fig. 5e) and SUM102 (Fig. 5f) structures at day 21. If CAFs were embedded within the rBM containing DQ-collagen IV and DCIS cells seeded on top, MEP-CM was observed to reduce the size of DCIS structures even in the presence of tumor-promoting CAFs (Additional file 8: Video S3 and Additional file 9: Video S4). Again, this is consistent with factors secreted by MEPs counteracting paracrine signaling between CAFs and DCIS. To verify that this reduction was not due to an effect on cell viability, we performed a live/dead assay on DCIS cells treated with MEP-CM for 16 days and confirmed that the DCIS cells remained viable (Additional file 10: Figure S6).

**Additional file 8: Video S3.** CAFs increase dysplastic phenotype of DCIS structures in 8-day cocultures. 3D reconstructions of 8-day MAME cultures with the following modifications: MCF10.DCIS-lenti-RFP (DCIS, *red*) cells were seeded on rBM containing embedded WS-12T-lenti-YFP (CAFs, *pseudocolored fuchsia*) and DQ-collagen IV (dDQ-IV, *green*). Merged fluorescence appears *white*. Grid unit = 45 μm. (MOV 10185 kb)




**Additional file 9: Video S4.** MEP-CM reduce dysplastic phenotype of DCIS-CAF structures in 8-day cocultures. 3D reconstructions of 8-day MAME cultures with the following modifications: MCF10.DCIS-lenti-RFP (DCIS, *red*) cells were seeded in the presence of MEP-CM on rBM containing embedded WS-12T-lenti-YFP (CAFs, *pseudocolored fuchsia*) and DQ-collagen IV (dDQ-IV, *green*). Merged fluorescence appears *white*. Grid unit = 45 μm. (MOV 12850 kb)


### rPAI-1 or ATN-617, a uPAR blocking antibody, reduces proteolysis and growth of DCIS structures

**Fig. 6:**
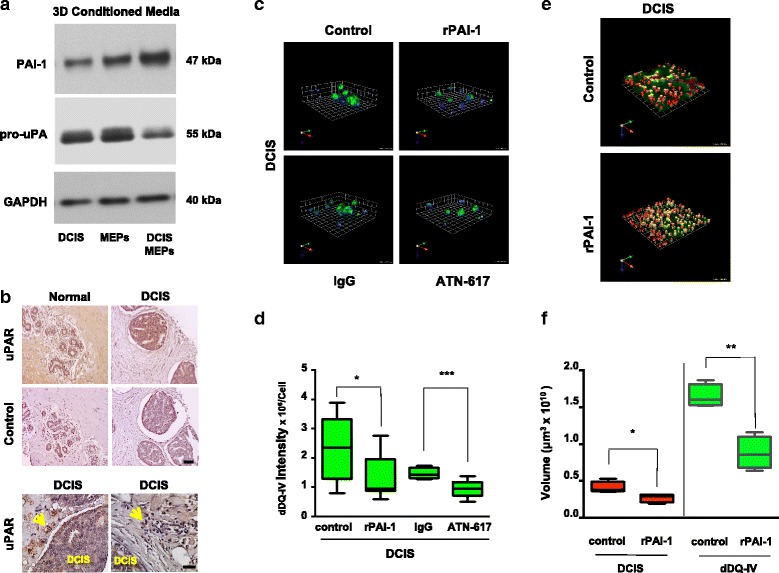
Analysis and targeting of the plasminogen activation pathway results in decreased extracellular matrix (ECM) degradation by ductal carcinoma in situ (DCIS) structures formed in mammary architecture and microenvironment engineering (MAME) cultures. **a** Media conditioned from 8-day 3D cultures of myoepithelial cells (MEPs) alone, DCIS cells alone, and DCIS-MEP cocultures were analyzed by immunoblotting for plasminogen activator inhibitor 1 (PAI-1) and pro-urokinase plasminogen activator (pro-uPA). Immunoblotting for glyceraldehyde 3-phosphate dehydrogenase (GAPDH) in cell lysates was used as a loading control. **b** Representative images from a tissue microarray containing adjacent normal and DCIS specimens were stained for human urokinase plasminogen activator receptor (uPAR) with ATN-617 antibody (5 μg/ml) (*top rows*) and preimmune immunoglobulin G (IgG) (control). Scale bar = 100 μm (*middle rows*). In DCIS specimens imaged at a higher magnification, stromal cells (*arrows*) can be seen to exhibit strong staining for uPAR. Scale bar = 50 μm (*bottom rows*). All sections were counterstained with hematoxylin. **c** MCF10.DCIS (DCIS) cells were seeded in reconstituted basement membrane (rBM) overlay cultures containing dye-quenched collagen IV (DQ-collagen IV) in the absence (control) or presence of 250 nM human recombinant plasminogen activator inhibitor 1 (rPAI-1), preimmune IgG (IgG), or 10 μg/ml uPAR blocking antibody (ATN-617) and imaged live at day 4. Representative angled views of 3D reconstructions of DCIS structures illustrate nuclei (*blue*) and DQ-collagen IV degradation products (dDQ-IV, *green*). One grid unit = 45 μm. **d** Intensity of dDQ-IV per cell was quantified from a minimum of three independent experiments using Volocity software (*n* = 8–14). * *p* ≤ 0.05 and *** *p* ≤ 0.0005 as determined by unpaired *t* test, two-sided. MCF10.DCIS-lenti-RFP cells (DCIS, *red*) were seeded into rBM overlay cultures containing DQ-collagen IV in the absence (control) or presence of rPAI-1 and imaged live at day 4. **e** Representative angled views of 3D reconstructions of DCIS (*red*) structures and associated dDQ-IV (*green*). One grid unit = 180 μm. **f** Volumes of DCIS structures (*red*) and dDQ-IV (*green*) in the absence (control) and presence of rPAI-1 were quantified from a minimum of three independent experiments using Volocity software (*n* = 4). * *p* ≤ 0.05 and ** *p* ≤ 0.005 as determined by unpaired *t* test, two-sided. Data are presented as box-and-whisker plots where the box represents the interquartile range and whiskers represent minimum and maximum values. Images are representative of at least three independent experiments. Additional results are shown in Additional file 11: Table S1, Additional file 12: Table S2, and Additional file 13: Table S3

The dramatic reductions in size and invasiveness of DCIS structures induced by MEP-CM prompted us to perform proteomic analyses on MEP-CM. In CM of MEPs grown in both 2D and 3D cultures, the highest protein scores were found for PAI-1, an inhibitor of plasminogen activation (Additional file 11: Table S1, Additional file 12: Table S2, and Additional file 13: Table S3). PAI-1 was also found to have the highest protein score in CM from DCIS-MEP cocultures, a score that was also greater than that of DCIS grown alone in 3D (Additional file 11: Table S1, Additional file 12: Table S2, and Additional file 13: Table S3). PAI-1 secretion by these cells was confirmed by immunoblotting (Fig. 6a), with the highest level of PAI-1 being secreted from DCIS-MEP cocultures. Among its multiple roles, PAI-1 is an inhibitor of uPA, a secreted serine protease involved in proteolytic cascades that promote tumorigenesis [56]. Our immunoblotting data revealed a slight decrease in secretion of pro-uPA (latent isoform) from DCIS-MEP cocultures (Fig. 6a). An increase in PAI-1 secretion and a decrease in pro-uPA secretion from DCIS-MEP cocultures are consistent with the observed reduction in DQ-collagen IV degradation and invasion in these cocultures, as well as with a role for the plasminogen pathway, which includes activation of plasminogen to plasmin by uPA.

Plasminogen activation and subsequent ECM proteolysis, processes linked to cancer progression and invasion, involve binding of uPA to its cell surface receptor uPAR [57]. Inhibition of uPA and its interactions with uPAR reduces tumor growth and invasion [58, 59]. Having demonstrated staining for uPAR in DCIS (Fig. 6b, *top row*) and associated stromal cells (Fig. 6b, *bottom row*), we compared the effects of both rPAI-1 and the uPAR blocking antibody ATN-617 [47] on degradation of DQ-collagen IV in MAME DCIS cultures. After 4 days in culture, degradation of DQ-collagen IV by the DCIS structures was significantly reduced by either rPAI-1 or ATN-617 (Fig. 6c and d). To further investigate the effects of PAI-1, we transduced DCIS cells with lenti-RFP so that volumes of DCIS structures and associated DQ-collagen IV degradation products could be measured. We found that rPAI-1 significantly reduced volumes of both DCIS structures and DQ-collagen IV degradation products (Fig. 6e and f). Our results are thus consistent with PAI-1 secreted by MEPs suppressing DCIS growth, blocking uPA binding to uPAR, and inactivating proteolytic networks involved in ECM remodeling and invasion.

### Blocking IL-6 reduces size of, and ECM degradation by, DCIS structures

**Fig. 7:**
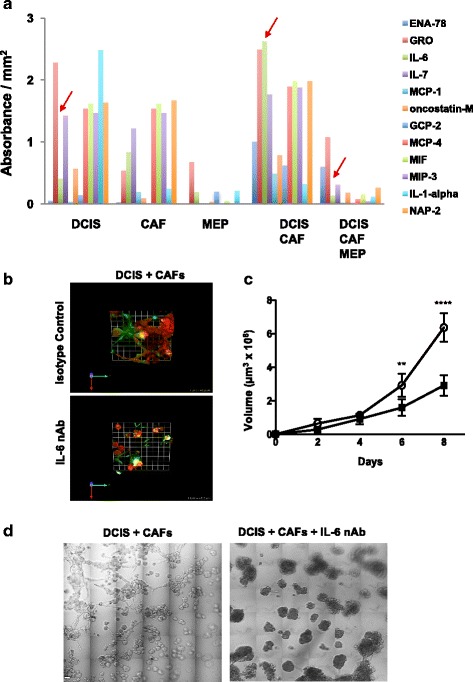
Targeting interleukin 6 (IL-6) reduces size and invasiveness of and extracellular matrix (ECM) degradation by ductal carcinoma in situ/cancer-associated fibroblast (DCIS-CAF) structures formed in mammary architecture and microenvironment engineering (MAME) cocultures. **a** Secretion of cytokines was assessed in 8-day conditioned media with a RayBio G5 human cytokine antibody array (RayBiotech, Norcross, GA, USA). Secretion of IL-6 was significantly elevated (*p* = 0.002 as determined by one-way analysis of variance [ANOVA]) when MCF10.DCIS (DCIS) cells were cocultured with WS-12T (CAFs), but it was not reduced when cocultured with myoepithelial cell (MEPs) or with CAFs plus MEPs (*arrows*). **b** MCF10.DCIS-lenti-RFP (DCIS) and WS-12T (CAFs) cells were seeded onto reconstituted basement membrane (rBM) overlaid with 2% rBM in the presence of isotype control or 100 ng/ml IL-6 neutralizing antibody (nAb) and imaged live at day 8. Representative en face views of 3D reconstructions of DCIS (*red*)-CAF (unlabeled) structures and associated degraded dye-quenched collagen IV (dDQ-IV; *green*) in MAME cultures; areas of colocalization appear *yellow-white*. One grid unit = 45 μm. **c** Volume of structures formed in MAME cultures of DCIS and CAFs in the presence of isotype control (*open circles*) or 100 ng/ml IL-6 nAb (*filled squares*) at days 2, 4, 6, and 8. Volume of structures was measured with Volocity software (*n* = 4). Data represent mean ± SD. ** *p* ≤ 0.01 and **** *p* ≤ 0.0001 as determined by two-way ANOVA. **d** Differential interference contrast images of MCF10.DCIS (DCIS) and WS-12T (CAFs) MAME cultures in the presence of isotype control or 100 ng/ml IL-6 nAb at day 8. Images are 36 contiguous tiled fields. Scale bar = 80 μm. Images are representative of at least three independent experiments. Additional results are shown in Additional file 14: Figure S7. *ENA-78* Epithelial-derived neutrophil-activating peptide 78, *GRO* Growth-related oncogene, *MCP* Monocyte chemoattractant protein, *GCP-2* Granulocyte chemotactic protein 2, *MIF* Macrophage migration inhibitory factor, *MIP-3* Macrophage inflammatory protein 3, *NAP-2* Neutrophil-activating protein 2

In the breast tumor microenvironment, fibroblasts promote growth and invasion through cytokine signaling [21, 60]. To assess if changes in morphology and growth of DCIS are due to modulation of cytokine secretion by CAFs, DCIS cells were grown either alone or with CAFs and/or MEPs for 8 days. The cytokines IL-6, epithelial-derived neutrophil-activating peptide 78﻿(ENA-78), and monocyte chemoattractant protein 1 (MCP-1) were found to be secreted at higher levels from DCIS-CAF cocultures than from DCIS cultures (Fig. 7a) and at reduced levels when MEPs were added to the DCIS-CAF cocultures (Fig. 7a). Because IL-6 was the cytokine most abundantly secreted from DCIS-CAF cocultures, we investigated whether the tumor-suppressing effects of MEPs on DCIS in the presence of CAFs (i.e., reduced cell growth, ECM proteolysis, and invasion) were a result of decreased IL-6 secretion. In the presence of neutralizing IL-6 antibodies, degradation of DQ-collagen IV was reduced in DCIS-CAF cocultures (Fig. 7b). Similar results were observed in MAME cocultures of SUM102 and CAFs (Additional file 14: Figure S7). To further elucidate the effect of IL-6 on DCIS growth, the volumes of DCIS-CAF structures were measured in the presence and absence of IL-6 neutralizing antibody at days 2, 4, 6, and 8. As expected, there was a significant reduction in volume of DCIS-CAF structures (Fig. 7c). Furthermore, blocking IL-6 reduced invasive outgrowths from the DCIS-CAF structures (Fig. 7d), effects similar to those observed in the presence of MEPs (Fig. 3b). Our data are consistent with an interplay between CAFs and MEPs in which CAF-secreted IL-6 drives progression and invasion of DCIS via a mechanism that can be attenuated by MEPs.

## Discussion

Premalignant DCIS is considered a nonobligate precursor to invasive carcinoma. Cellular mechanisms that promote the transition of DCIS to invasive carcinoma remain unclear [61]. Moreover, the lack of a robust molecular signature impedes the development of clinical tests to predict which DCIS lesions will progress to invasive carcinomas [4, 61]. This is further complicated by the histological and biological diversity of DCIS that influences the rate of progression, prognosis, and responses to specific therapies [62]. Studies identifying drivers of DCIS progression have been focused on stage-specific changes in the tumor microenvironment [4, 63, 64]. Authors of a recent report identified subtype-specific signatures that underline a role for the tumor microenvironment in predicting the transition of preinvasive to invasive breast cancer [65]. In the present study, we employed both mammary and renal xenograft mouse models to assess the effects of MEPs and CAFs on DCIS progression and a 3D pathomimetic MAME model of DCIS cultures/cocultures to study the mechanisms that drive these effects. We reveal that the tumor-suppressive effects of MEPs on DCIS are (1) linked to inhibition of uPA/uPAR-mediated proteolysis by PAI-1 and (2) can reduce the tumor-promoting effects of CAFs, in part by attenuating the signaling pathways involving the proinflammatory cytokine IL-6.

In normal breast tissue, MEPs are localized between the luminal epithelial cells and the basement membrane of the mammary ducts and alveoli. MEPs maintain proper organization and function of breast tissue by promoting epithelial cell polarity and facilitating expulsion of glandular secretions while preventing epithelial invasion into the surrounding ECM [10]. The loss of the MEP layer coincides with a high risk of DCIS progression into invasive carcinoma [66]. We show in both mammary and renal xenograft models that, in the presence of MEPs, DCIS xenografts were smaller, better organized, and exhibited less collagen deposition than xenografts of DCIS cells alone. This is consistent with results obtained in a subcutaneous DCIS xenograft [19]. Our MAME model, which is designed to recapitulate mammary architecture in vitro [30], also showed a lessened dysplastic phenotype of DCIS cells in the presence of MEPs that was accompanied by reduced DQ-collagen IV proteolysis, decreased secretion of pro-uPA, and increased secretion of PAI-1.

Pro-uPA is the secreted precursor of uPA, a serine protease involved in extracellular proteolytic networks encompassing many proteases (e.g., plasmin, MMPs, and cysteine proteases) that promote tumorigenesis, cell proliferation, invasion, and metastasis [56]. Activation of pro-uPA involves binding of the latent enzyme to its cell surface receptor, uPAR. Increased expression of uPA and uPAR has been linked to poor prognosis of breast cancer [56]. In areas of DCIS microinvasion, stromal myofibroblasts and macrophages stain for uPA, uPAR, and MMP-13, suggesting that these proteases work cooperatively in promoting the transition of DCIS to invasive carcinoma [67]. Using a 3D collagen I assay, myofibroblasts were shown to direct breast cancer cell motility in a plasminogen-dependent manner [68]. We localized uPAR to both tumor and stromal cells in DCIS tissue and demonstrated that blocking uPAR in DCIS cells decreased DQ-collagen IV degradation in vitro. Our data thus support a role for uPA/uPAR during the transition of DCIS into invasive carcinoma, a mechanism that may be attenuated by the high levels of PAI-1 secreted by MEPs in preinvasive DCIS lesions.

The role of PAI-1 in breast cancer is multifaceted and stage-specific. Increased PAI-1 alone or in combination with high uPA levels in breast cancer has been linked to poor prognosis [69, 70]. Increased PAI-1 expression has also been observed in MEPs from high-grade DCIS [71, 72]. On one hand, PAI-1 in high-grade DCIS-associated MEPs may alter cell-matrix adhesion between MEPs and the underlying basement membrane by disrupting uPAR binding to basement membrane proteins [73]. Our studies, on the other hand, suggest a protective role for PAI-1 secreted by normal MEPs in inhibiting uPA/uPAR proteolytic pathways involved in ECM degradation. Hence, a dual role for PAI-1 secreted by MEPs, first as an inhibitor of uPA, ECM degradation, and tumor invasion in normal breast and then as a promoter of cell detachment to facilitate migration and invasion in high-grade DCIS, would be consistent with the hypothesis that changes in the tumor microenvironment are major contributors to DCIS progression. Indeed, changes in DCIS-associated MEPs have been shown to result in increased expression of proangiogenic and invasive genes and ECM-degrading proteases (e.g., MMP-2; MMP-14; and cathepsins F, K, and L) [4, 19, 74]. Moreover, disruption of signaling pathways that are essential to MEP differentiation and mediated by transforming growth factor β, Hedgehog, cell adhesion, and p63 also results in loss of MEPs and accelerated progression of DCIS to invasive carcinoma [19]. Tumor-associated MEPs are also deficient in their ability to direct cell polarity of luminal epithelial cells [75]. These changes in MEPs during tumorigenesis likely alter the paracrine interactions between MEPs and stromal cells such as CAFs to facilitate tumor invasion.

Numerous studies have shown that CAFs enhance tumor growth and invasion by secretion of growth factors, cytokines, and proteases [16, 17]. In the present study, we show that in the presence of CAFs, orthotopic and renal capsule DCIS xenografts were larger and exhibited more collagen deposition, a stromal biomarker of breast cancer progression [76]. In our hands, adding CAFs to 3D DCIS MAME cultures resulted in larger structures with more invasive outgrowths and increased DQ-collagen IV degradation. Previous reports using both in vivo and in vitro DCIS models showed that CAFs induce an invasive DCIS phenotype in parallel with an increase in MMP-14 expression and MMP-9 activity [18, 19]. Interestingly, we show that the tumor-promoting actions of CAFs on DCIS cells could be attenuated by MEPs both in vivo and in vitro. Further analysis using our in vitro MAME model revealed that MEPs significantly decreased IL-6 secretion from DCIS-CAF cocultures. We previously reported that DCIS proliferation and ECM proteolysis, migration, and invasion are increased by normal fibroblasts induced to secrete HGF [20] and CAFs secreting IL-6 [21]. Secretion of HGF from these normal fibroblasts was correlated with an increase in uPA and uPAR secretion from DCIS cells [20]. Moreover, paracrine IL-6 signaling between DCIS cells and CAFs promotes tumor cell growth and migration in part through cathepsin B-mediated ECM degradation [21]. A role for cathepsin B is further supported by studies showing that suppression of cystatin A, an endogenous inhibitor of cathepsin B, increases progression of DCIS to invasive carcinoma [77]. Previous studies have also shown a tumor-promoting role for CAFs via IL-6 secretion [23] and an association between IL-6 secretion and upregulation of ECM-degrading enzymes (e.g., cathepsin B, MMPs, and uPA) [21, 24–27]. Our data suggest that MEPs prevent the tumor-promoting actions of CAFs by blocking secretion of proinflammatory factors, including IL-6, into the tumor microenvironment.

## Conclusions

**Fig. 8:**
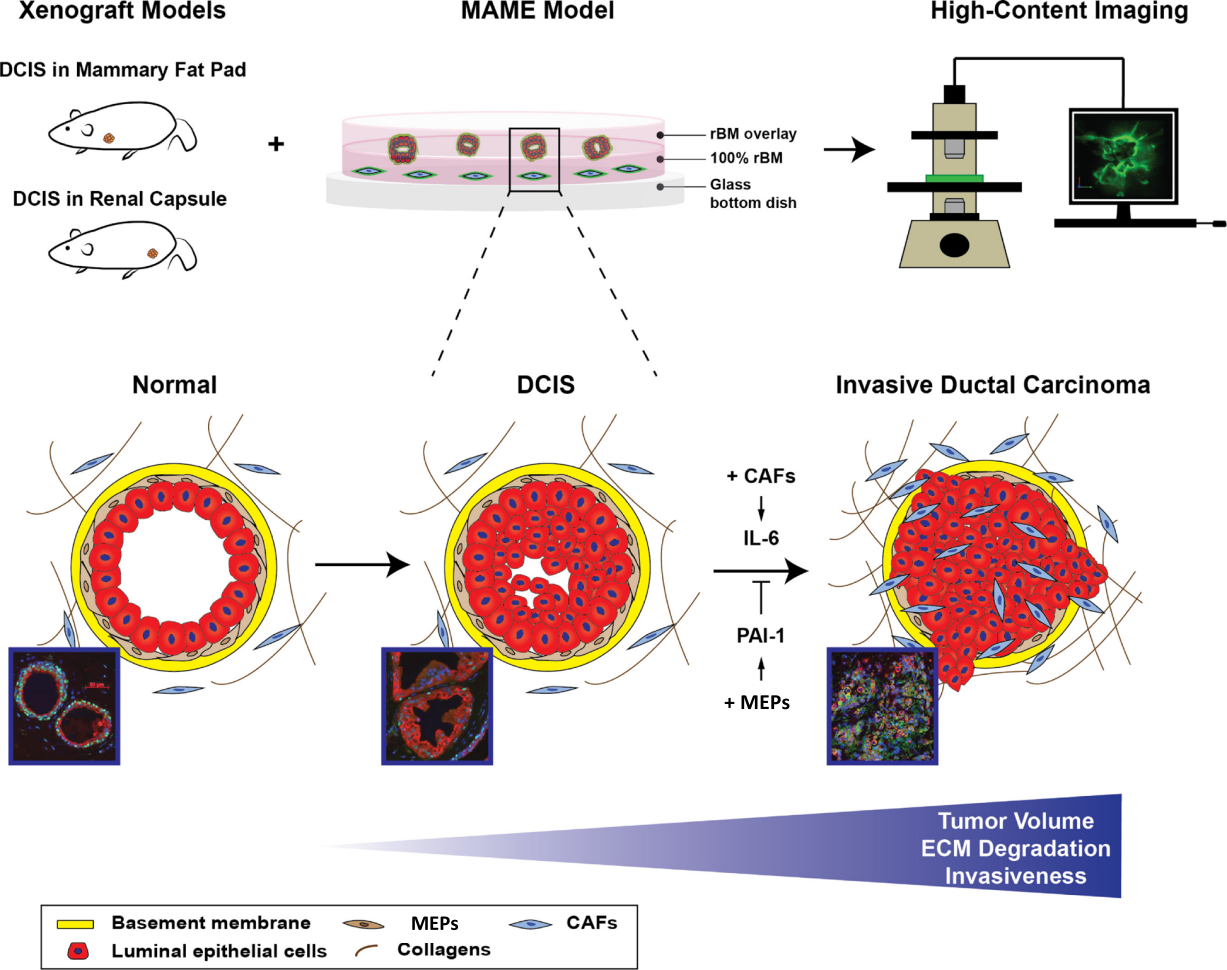
A schematic diagram illustrating the use of 3D pathomimetic mammary architecture and microenvironment engineering (MAME) cultures to identify a divergent interplay between tumor-suppressive myoepithelial cells (MEPs) and tumor-promoting cancer-associated fibroblasts (CAFs) that involves plasminogen activator inhibitor 1 (PAI-1), urokinase plasminogen activator/urokinase plasminogen activator receptor (uPA/uPAR), and interleukin 6 (IL-6) and alters the cellular and molecular phenotype of ductal carcinoma in situ (DCIS). *ECM* Extracellular matrix, *rBM* Reconstituted basement membrane

Using our 3D pathomimetic MAME cultures, we identified a divergent interplay between tumor-suppressive MEPs and tumor-promoting CAFs that involves PAI-1, uPA/uPAR, and IL-6 and that alters the cellular and molecular phenotype of DCIS (Fig. 8). We propose that these interactions evolve during DCIS progression through changes in the tumor microenvironment that promote invasion and metastasis. In a 3D compartmentalized microfluidic model in which DCIS cells and fibroblasts were cocultured separately, factors secreted by the fibroblasts were shown to change the morphology and invasiveness of DCIS structures and their remodeling of ECM collagen [32]. Using our MAME models, we have previously shown that changes in the noncellular microenvironment also affect the invasive potential of breast carcinoma cells. For example, MAME cultures of MDA-MB-231 cells maintained at a slightly acidic pH (6.8), a pH comparable to that in the microenvironment of solid tumors, exhibit highly increased degradation of type IV collagen [34]. We have also used MAME as a preclinical model to test the effects of cabozantinib on the tumor-promoting interactions between different triple-negative breast cancer cells, representing various molecular subtypes of the disease, and CAFs and normal fibroblasts overexpressing HGF [78]. One advantage of using such pathomimetic models to study breast cancer progression is the control over spatial and physical parameters of the tumor microenvironment without disturbance of the 3D heterotypic cultures. Another is the ability to examine differential effects of therapies on multiple 3D structures that may be consistent with tumor heterogeneity. These pathomimetic models could also be adapted for precision medicine using DCIS patient-derived cells and applied to studies analyzing drug resistance and screening of novel therapeutic approaches.

## Additional files

Additional file 1: Figure S1. Characterization of DCIS xenografts and MEPs using basal markers and laminin-332. MCF10.DCIS (DCIS), N1ME (MEPs), and/or WS-12T (CAFs) cells were implanted under the renal capsule or orthotopically within the mammary fat pad of female SCID mice and evaluated after 8 weeks. Representative (**a**) orthotopic and (**b**) renal xenografts immunostained for αSMA (*green*, cytoplasmic staining) and CK14 (*red*, cytoplasmic staining), p63 (*red*, nuclear staining), and Hoechst 33342 (*blue nuclei*). *Arrows* represent areas of colocalization. Original magnification × 60. **c** Immunoblotting of MEPs shows expression of αSMA, CD10, and CK17 in N1ME cells. **d** Representative orthotopic and renal xenografts immunostained for laminin-332. Scale bar = 100 μm. (PDF 492 kb)

Additional file 4: Figure S2. Laminin-332 staining in normal human breast and DCIS. Representative images are shown from a tissue microarray containing adjacent normal and DCIS specimens and stained with human laminin-332 antibody (10 μg/ml). **a** Adjacent sections for normal and DCIS were processed using preimmune IgG (control). Scale bar = 100 μm. **b** Higher-magnification images show diffuse staining for laminin-332 in DCIS cells. Scale bar = 50 μm. All sections were counterstained with hematoxylin. (PDF 1544 kb)

Additional file 5: Figure S3. MEPs reduce invasive outgrowths from DCIS structures formed in MAME cultures. MCF10.DCIS-lenti-RFP cells (DCIS) were seeded into MAME cultures alone or with N1ME cells (MEPs) and imaged live at day 16. 3D reconstructions of Z-stack images of DCIS (*red*) structures (*top row*) and DCIS (*red*) plus MEP structures (*bottom row*) are shown (*green* represents DQ-collagen IV degradation products). One grid unit = 90 μm. Reconstructions are shown in *left column* in an en face view and at various angles of view in the other columns. In the *top row*, the *arrows* point to the same invasive outgrowth in each image. (PDF 2002 kb)

Additional file 6: Figure S4. MEPs reduce size of DCIS structures formed in MAME cultures. Representative angled and en face views of 3D reconstructions of 8- and 21-day MAME cultures of MCF10.DCIS-lenti-RFP (DCIS, *red*) cells seeded alone (*top row*, grid unit = 92 μm) or in coculture with N1ME (MEPs, unlabeled; *second row*, grid unit = 90 μm), WS-12T-lenti-YFP (CAFs, *pseudocolored fuchsia*; third row, grid unit = 92 μm), or both CAFs and MEPs (*bottom row*, grid unit = 90 μm) in an rBM overlay culture containing DQ-collagen IV (dDQ-collagen IV, *green*). Areas of dDQ-collagen IV on surface of DCIS structures appear *yellow*. (PDF 654 kb)

Additional file 7: Figure S5. MEP-conditioned media (MEP-CM) reduce size of DCIS structures formed in MAME cultures. DCIS cells were seeded in rBM overlay cultures containing DQ-collagen IV in the absence (control) or presence of MEP-conditioned media (MEP-CM) and imaged live at day 8. DIC images are 16 contiguous tiled fields of structures formed by two DCIS cell lines: MCF10.DCIS-lentiRFP (DCIS; **a**, *top rows*; scale bars = 90 μm) and SUM102-lentiRFP (SUM102; **b**, *top rows*; scale bars, 180 μm). Fluorescent images are en face views of 3D reconstructions of DCIS (**a**, *bottom rows*) and SUM102 (**b**, *bottom rows*) structures (*red*) and associated dDQ-IV (*green*). One grid unit = 45 μm. (PDF 1373 kb)

Additional file 10: Figure S6. MEP-conditioned media (MEP-CM) are not cytotoxic to DCIS structures. MCF10.DCIS (DCIS) cells were seeded into rBM overlay cultures in the absence (control) or presence of MEP-conditioned media (MEP-CM). A live/dead assay was performed on 16-day cultures; *green* and *red* represent live and dead cells, respectively. (PDF 119 kb)

Additional file 11: Table S1. Comparative proteomic analysis of conditioned media from 2D and 3D MEP and DCIS cultures. Protein scores >28 indicate identity or extensive homology (*p* ≤ 0.05). *ND* Not detected. (PDF 17 kb)

Additional file 12: Table S2. Proteomic analysis of conditioned media from 2D MEP cultures. (PDF 50 kb)

Additional file 13: Table S3. Proteomic analysis of conditioned media from 3D MEP and DCIS cultures. (PDF 57 kb)

Additional file 14: Figure S7. Targeting IL-6 reduces size and invasiveness of and ECM degradation by SUM102-CAF structures formed in MAME cultures. SUM102-lentiRFP and WS-12T (CAFs) were seeded onto rBM overlaid with 2% rBM in the presence of isotype control or 100 ng/ml IL-6 neutralizing antibody (IL-6 nAb) and imaged live at day 8. Representative en face views of 3D reconstructions of SUM102 (*red*)-CAF (unlabeled) structures and associated dDQ-IV (*green*) in MAME cultures. One grid unit = 45 μm. (PDF 278 kb)

## Abbreviations

ANOVA: Analysis of variance
CAF: Cancer-associated fibroblast
CK: Cytokeratin
DCIS: Ductal carcinoma in situ
dDQ-IV: Degraded dye-quenched collagen IV
DIC: Differential interference contrast
DQ-collagen IV: Dye-quenched collagen IV
ECM: Extracellular matrix
ENA-78: Epithelial-derived neutrophil-activating peptide 78
GAPDH: Glyceraldehyde 3-phosphate dehydrogenase
GCP-2: Granulocyte chemotactic protein 2
GRO: Growth-related oncogene
H&E: Hematoxylin and eosin
HGF: Hepatocyte growth factor
IDC: Invasive ductal carcinoma
IgG: Immunoglobulin G
IL-6: Interleukin 6
MAME: Mammary architecture and microenvironment engineering
MCP: Monocyte chemoattractant protein
MEGM: Mammary epithelial growth medium
MEP: Myoepithelial cell
MEP-CM: Myoepithelial cell-conditioned media
MIF: Macrophage migration inhibitory factor
MIP-3: Macrophage inflammatory protein 3
MMP: Matrix metalloprotease
nAb: Neutralizing antibody
NAP-2: Neutrophil-activating protein 2
NLO: Nonlinear optical
PAI-1: Plasminogen activator inhibitor 1
rBM: Reconstituted basement membrane
RFP: Red fluorescent protein
SCID: Severe combined immunodeficiency
uPA: Urokinase plasminogen activator
uPAR: Urokinase plasminogen activator receptor
YFP: Yellow fluorescent protein
αSMA: α-Smooth muscle actin
