# Supplementary figures and images for: Pathomimetic avatars reveal divergent roles of microenvironment in invasive transition of ductal carcinoma in situ
Source: Breast Cancer Res. 2017 May 15;19:56. doi: 10.1186/s13058-017-0847-0 (PMC5433063; doi:10.1186/s13058-017-0847-0)

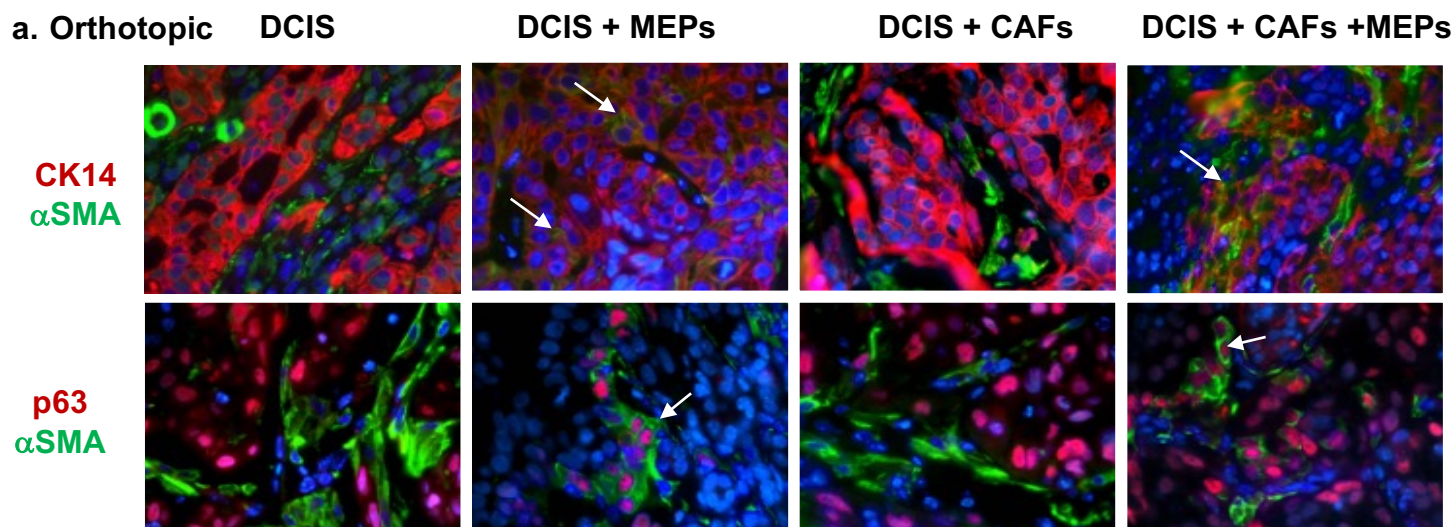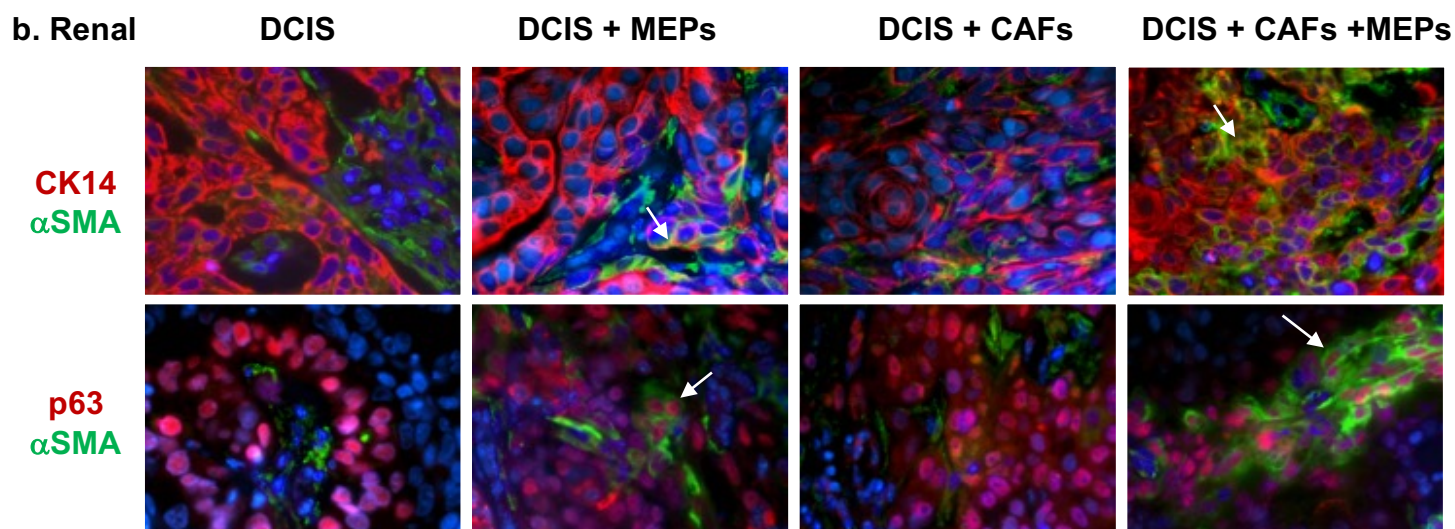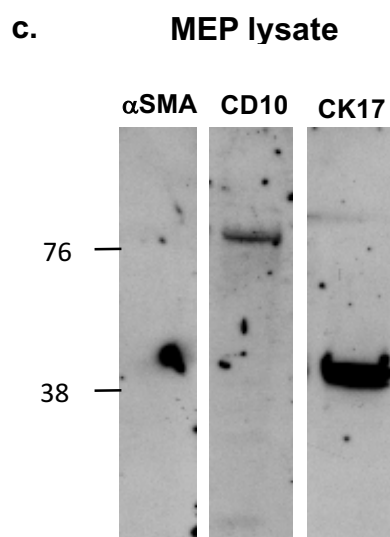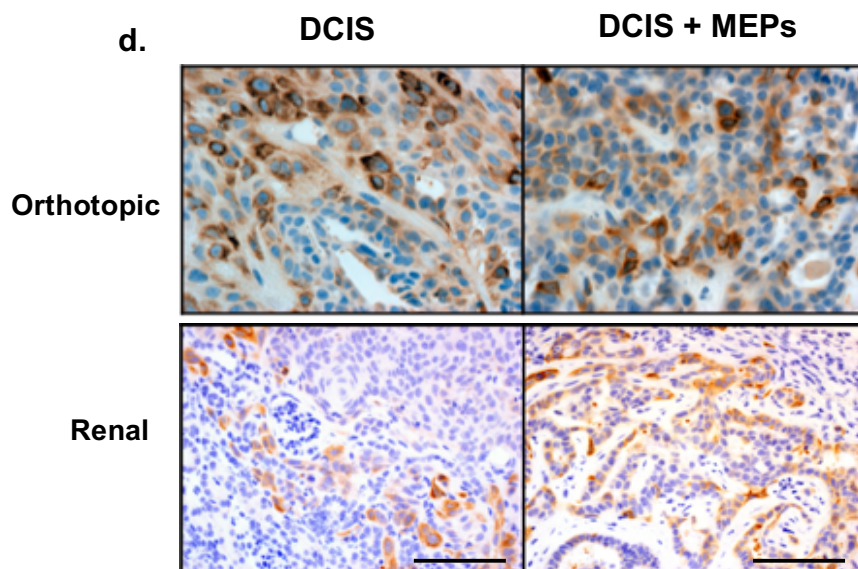

Additional File 1: Figure S1

Supplement: Supplementary file 5 — Characterization of DCIS xenografts and MEPs using basal markers and laminin-332. MCF10.DCIS (DCIS), N1ME (MEPs), and/or WS-12T (CAFs) cells were implanted under the renal capsule or orthotopically within the mammary fat pad of female SCID mice and evaluated after 8 weeks. Representative (a) orthotopic and (b) renal xenografts immunostained for αSMA (green, cytoplasmic staining) and CK14 (red, cytoplasmic staining), p63 (red, nuclear staining), and Hoechst 33342 (blue nuclei). Arrows represent areas of colocalization. Original magnification × 60. c Immunoblotting of MEPs shows expression of αSMA, CD10, and CK17 in N1ME cells. d Representative orthotopic and renal xenografts immunostained for laminin-332. Scale bar = 100 μm. (PDF 492 kb) [file 13058_2017_847_MOESM1_ESM.pdf]

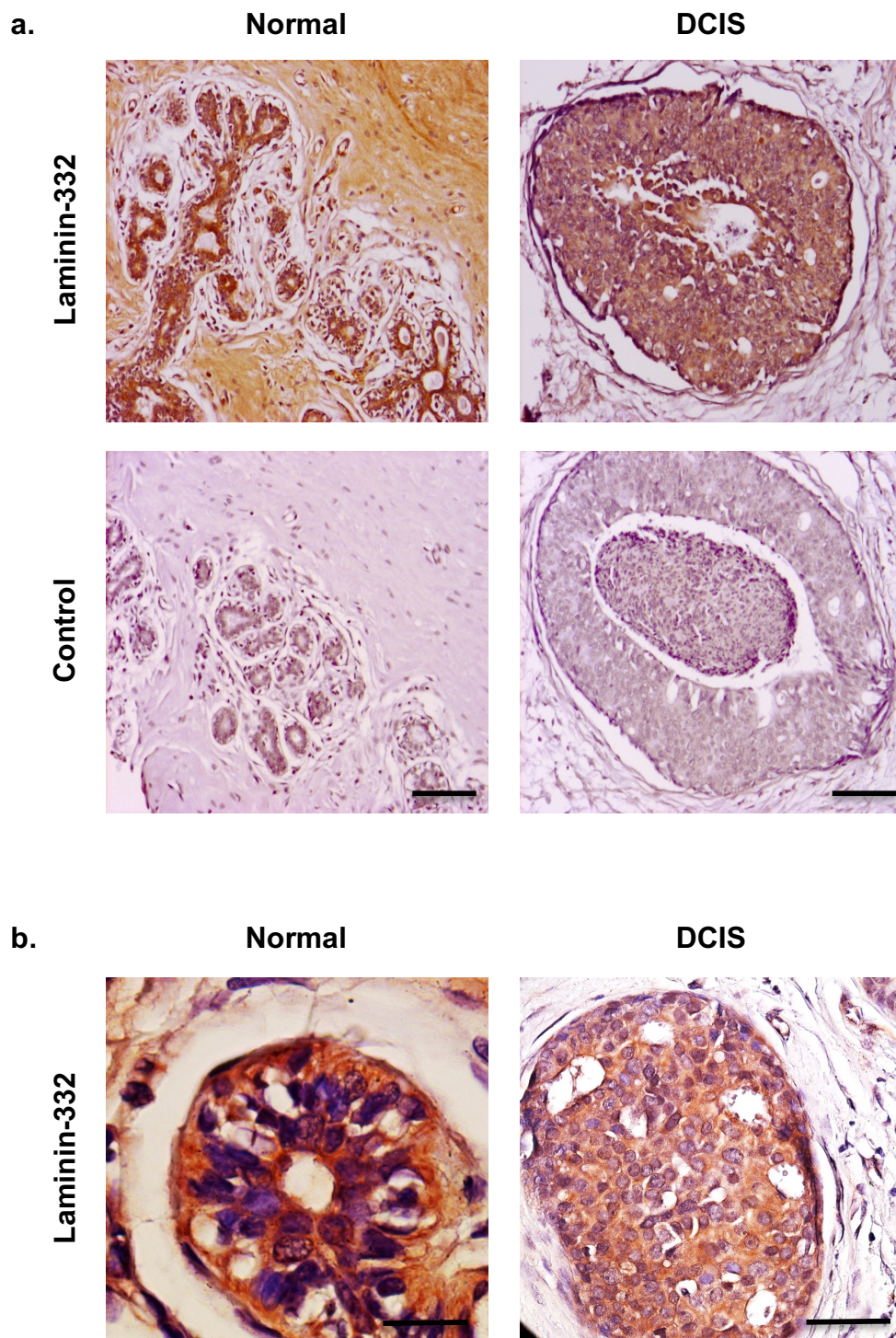

**Additional File 4: Figure S2**

Supplement: Supplementary file 6 — Laminin-332 staining in normal human breast and DCIS. Representative images are shown from a tissue microarray containing adjacent normal and DCIS specimens and stained with human laminin-332 antibody (10 μg/ml). a Adjacent sections for normal and DCIS were processed using preimmune IgG (control). Scale bar = 100 μm. b Higher-magnification images show diffuse staining for laminin-332 in DCIS cells. Scale bar = 50 μm. All sections were counterstained with hematoxylin. (PDF 1544 kb) [file 13058_2017_847_MOESM4_ESM.pdf]

## *En Face View*

## Angled Views

DCIS

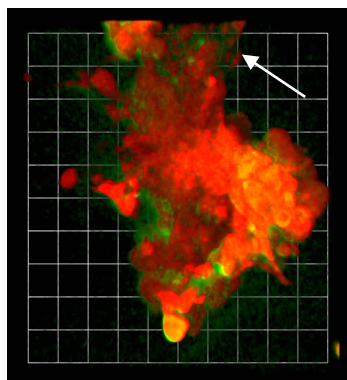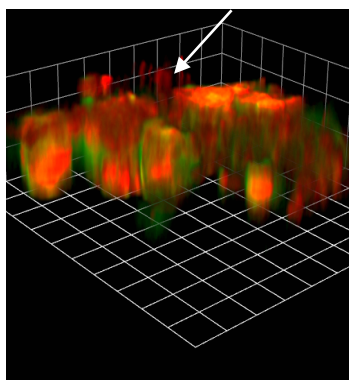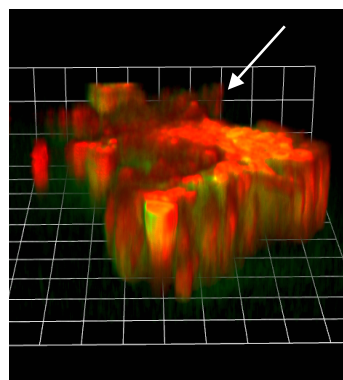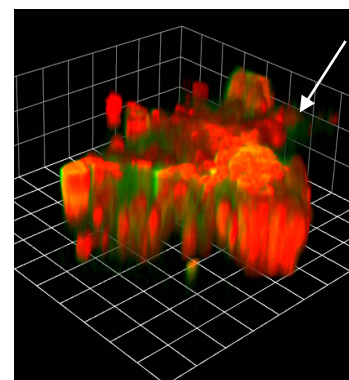

DCIS + MEPS

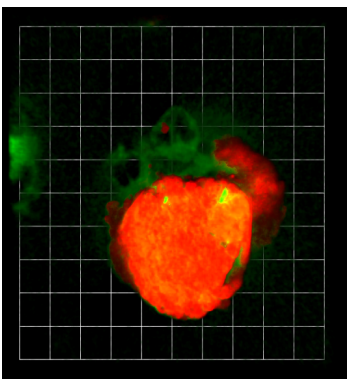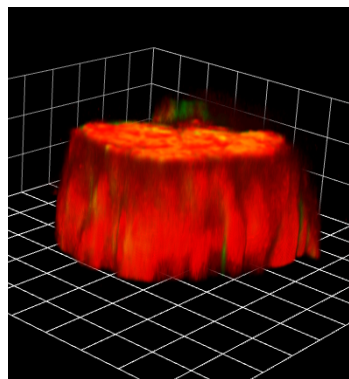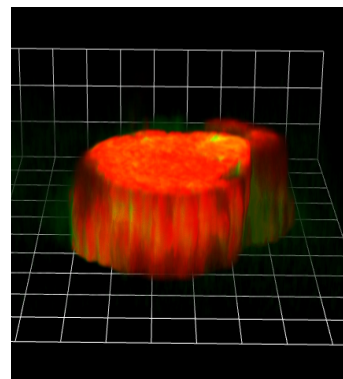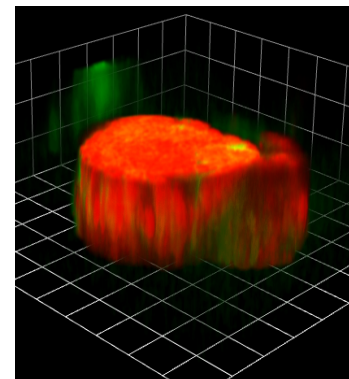

Supplement: Supplementary file 7 — MEPs reduce invasive outgrowths from DCIS structures formed in MAME cultures. MCF10.DCIS-lenti-RFP cells (DCIS) were seeded into MAME cultures alone or with N1ME cells (MEPs) and imaged live at day 16. 3D reconstructions of Z-stack images of DCIS (red) structures (top row) and DCIS (red) plus MEP structures (bottom row) are shown (green represents DQ-collagen IV degradation products). One grid unit = 90 μm. Reconstructions are shown in left column in an en face view and at various angles of view in the other columns. In the top row, the arrows point to the same invasive outgrowth in each image. (PDF 2002 kb) [file 13058_2017_847_MOESM5_ESM.pdf]

## DCIS

**a.**

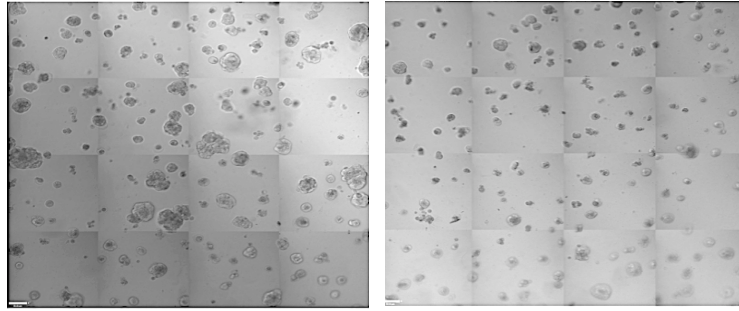

**8 days**

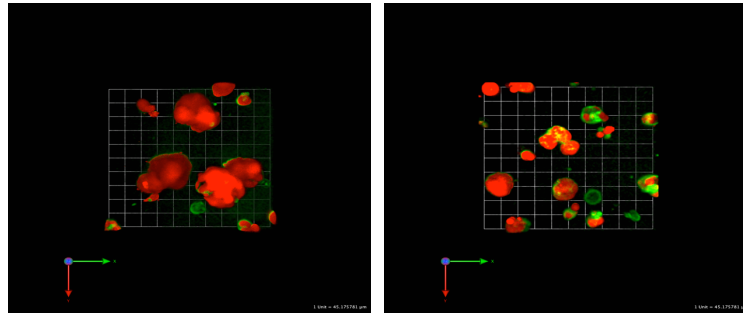

**Control**

**MEP-CM**

## SUM 102

**b.**

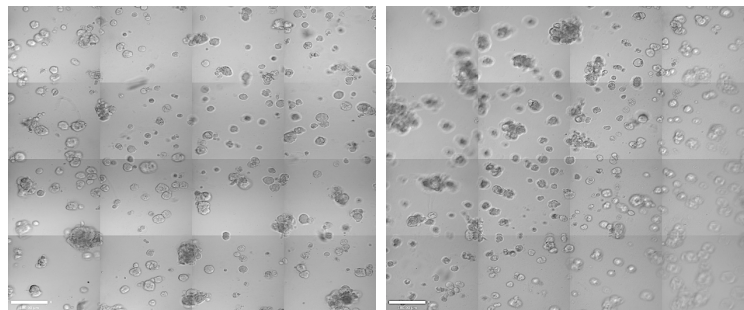

**8 days**

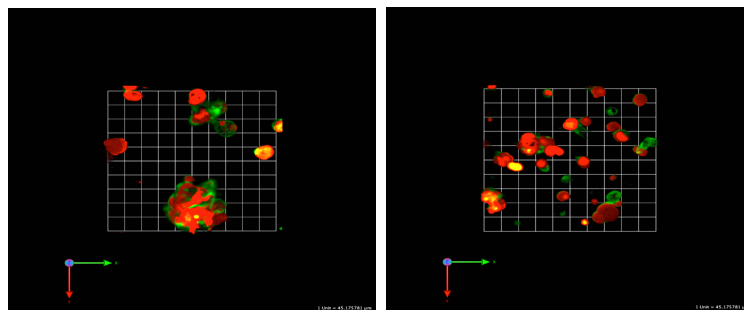

**Control**

**MEP-CM**

Supplement: Supplementary file 9 — MEP-conditioned media (MEP-CM) reduce size of DCIS structures formed in MAME cultures. DCIS cells were seeded in rBM overlay cultures containing DQ-collagen IV in the absence (control) or presence of MEP-conditioned media (MEP-CM) and imaged live at day 8. DIC images are 16 contiguous tiled fields of structures formed by two DCIS cell lines: MCF10.DCIS-lentiRFP (DCIS; a, top rows; scale bars = 90 μm) and SUM102-lentiRFP (SUM102; b, top rows; scale bars, 180 μm). Fluorescent images are en face views of 3D reconstructions of DCIS (a, bottom rows) and SUM102 (b, bottom rows) structures (red) and associated dDQ-IV (green). One grid unit = 45 μm. (PDF 1373 kb) [file 13058_2017_847_MOESM7_ESM.pdf]

Live / Dead

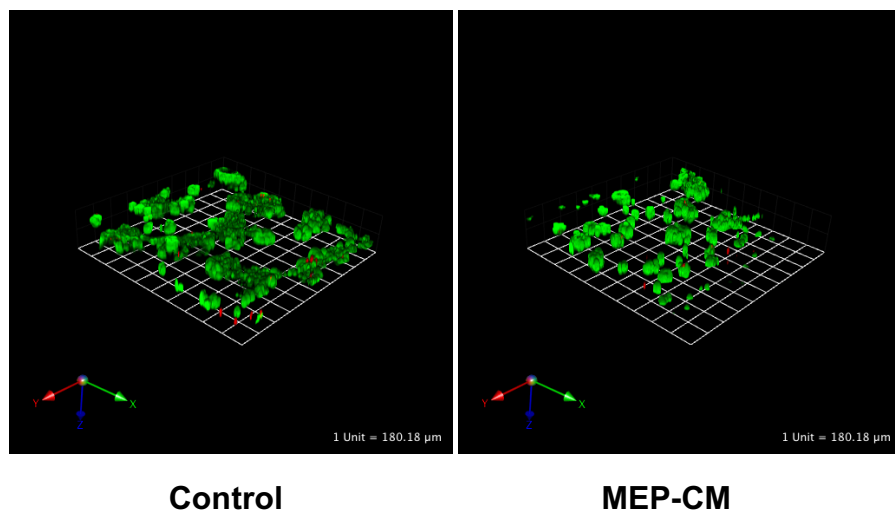

Supplement: Supplementary file 10 — MEP-conditioned media (MEP-CM) are not cytotoxic to DCIS structures. MCF10.DCIS (DCIS) cells were seeded into rBM overlay cultures in the absence (control) or presence of MEP-conditioned media (MEP-CM). A live/dead assay was performed on 16-day cultures; green and red represent live and dead cells, respectively. (PDF 119 kb) [file 13058_2017_847_MOESM10_ESM.pdf]

## SUM102 + CAFs

Isotype Control

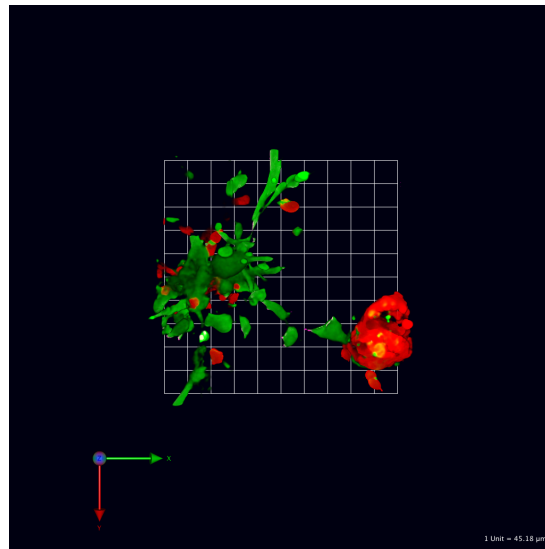

IL-6nAb

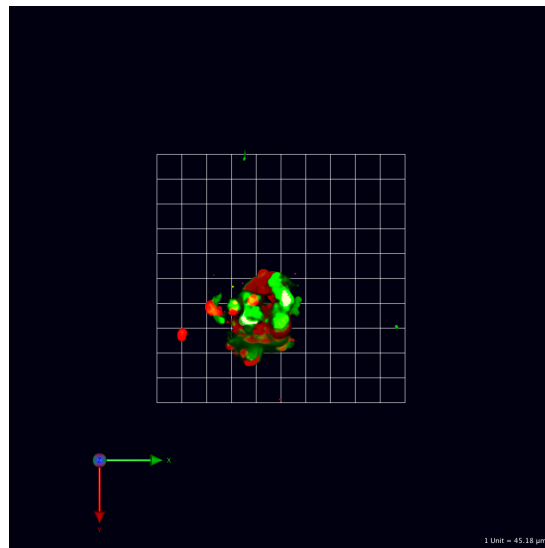

Supplement: Supplementary file 14 — Targeting IL-6 reduces size and invasiveness of and ECM degradation by SUM102-CAF structures formed in MAME cultures. SUM102-lentiRFP and WS-12T (CAFs) were seeded onto rBM overlaid with 2% rBM in the presence of isotype control or 100 ng/ml IL-6 neutralizing antibody (IL-6 nAb) and imaged live at day 8. Representative en face views of 3D reconstructions of SUM102 (red)-CAF (unlabeled) structures and associated dDQ-IV (green) in MAME cultures. One grid unit = 45 μm. (PDF 278 kb) [file 13058_2017_847_MOESM14_ESM.pdf]
