# Supplementary material for: Pathomimetic avatars reveal divergent roles of microenvironment in invasive transition of ductal carcinoma in situ
Source: Breast Cancer Res. 2017 May 15;19:56. doi: 10.1186/s13058-017-0847-0 (PMC5433063; doi:10.1186/s13058-017-0847-0)
Supplement: Supplementary file 8 — MEPs reduce size of DCIS structures formed in MAME cultures. Representative angled and en face views of 3D reconstructions of 8- and 21-day MAME cultures of MCF10.DCIS-lenti-RFP (DCIS, red) cells seeded alone (top row, grid unit = 92 μm) or in coculture with N1ME (MEPs, unlabeled; second row, grid unit = 90 μm), WS-12T-lenti-YFP (CAFs, pseudocolored fuchsia; third row, grid unit = 92 μm), or both CAFs and MEPs (bottom row, grid unit = 90 μm) in an rBM overlay culture containing DQ-collagen IV (dDQ-collagen IV, green). Areas of dDQ-collagen IV on surface of DCIS structures appear yellow. (PDF 654 kb) [file 13058_2017_847_MOESM6_ESM.pdf]

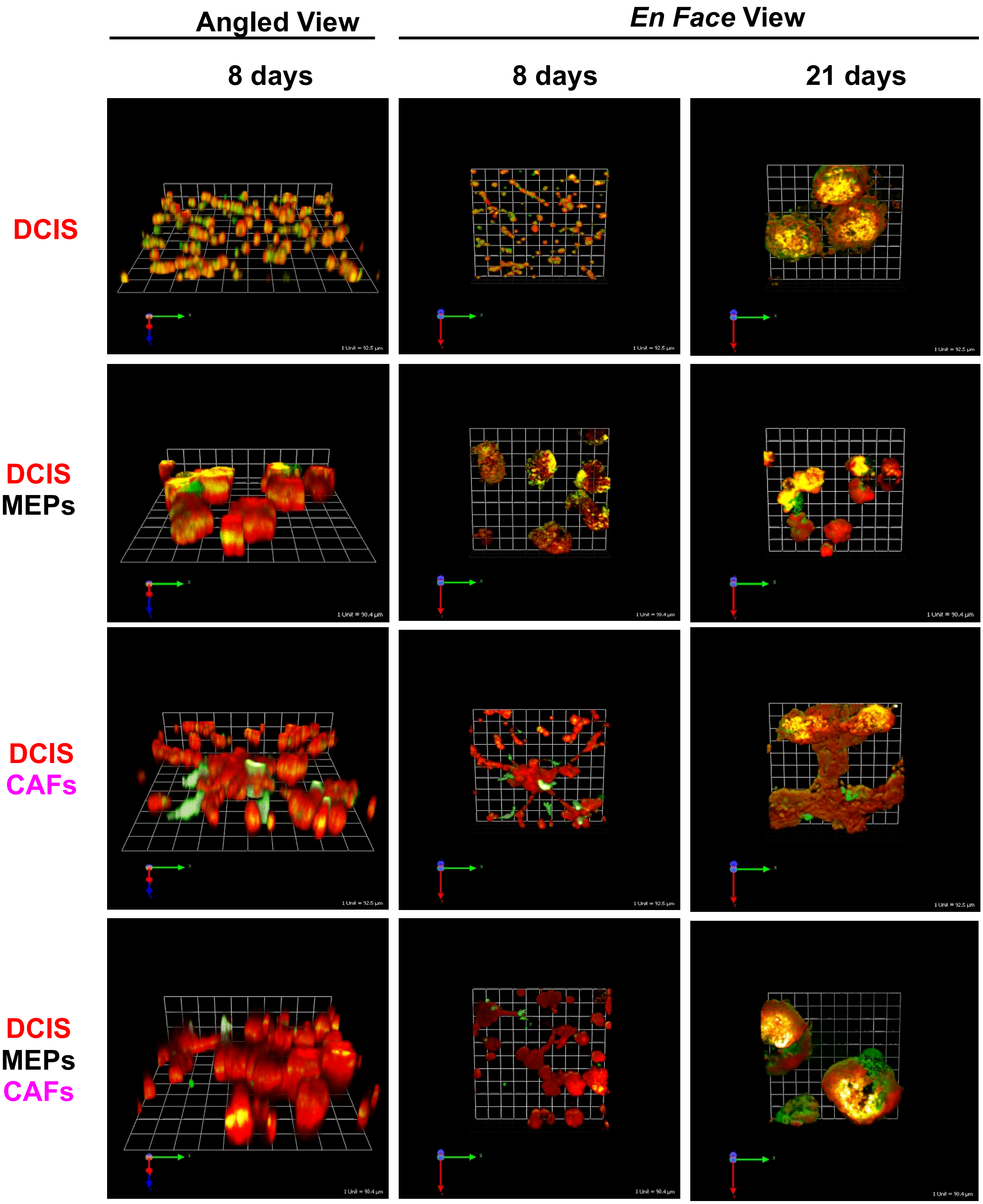

Additional File 6: Figure S4
