# Supplementary material for: Pathomimetic avatars reveal divergent roles of microenvironment in invasive transition of ductal carcinoma in situ
Source: Breast Cancer Res. 2017 May 15;19:56. doi: 10.1186/s13058-017-0847-0 (PMC5433063; doi:10.1186/s13058-017-0847-0)
Supplement: Supplementary file 11 — Comparative proteomic analysis of conditioned media from 2D and 3D MEP and DCIS cultures. Protein scores >28 indicate identity or extensive homology (p ≤ 0.05). ND Not detected. (PDF 17 kb) [file 13058_2017_847_MOESM11_ESM.pdf]

Additional File 11: Table S1. Comparative Proteomic Analysis of Conditioned Media from 2D and 3D MEP and DCIS Cultures. Protein scores >28 indicate identity or extensive homology ($p \leq 0.05$). ND, not detected.

Protein Name	Protein Score			
	2D Cultures	3D Cultures		
	MEPs	DCIS	MEPs	DCIS + MEPs
**PAI-1**	249	41	77	146
**α-enolase**	159	ND	28	99
**Fibronectin**	155	ND	35	ND
**RabGD1α**	102	ND	ND	ND
**Laminin 332**	88	ND	ND	24
