# Supplementary material for: Pathomimetic avatars reveal divergent roles of microenvironment in invasive transition of ductal carcinoma in situ
Source: Breast Cancer Res. 2017 May 15;19:56. doi: 10.1186/s13058-017-0847-0 (PMC5433063; doi:10.1186/s13058-017-0847-0)
Supplement: Supplementary file 12 — Proteomic analysis of conditioned media from 2D MEP cultures. (PDF 50 kb) [file 13058_2017_847_MOESM12_ESM.pdf]

Additional File 12: Table S2. Proteomic Analysis of Conditioned Media from 2D MEP Cultures.

Identified Proteins	UniProt Entry name	Molecular Weight	2D	
			Media (control)	MEPs
			# of peptides	
Serum albumin	ALBU_HUMAN	69 kDa	2	4
Keratin, type II cytoskeletal 2 epidermal	K22E_HUMAN	66 kDa	4	0
Hemoglobin subunit alpha	HBA_HUMAN	15 kDa	2	3
Hemoglobin subunit gamma-1	HBG1_HUMAN	16 kDa	1	2
Heat shock protein HSP 90-alpha	HS90A_HUMAN	85 kDa	4	1
Fibronectin	FINC_HUMAN	263 kDa	0	5
Alpha-enolase	ENOA_HUMAN	47 kDa	2	2
Ubiquitin-60S ribosomal protein L40	RL40_HUMAN	15 kDa	2	2
Triosephosphate isomerase	TPIS_HUMAN	31 kDa	1	3
Tubulin alpha-1B chain	TBA1B_HUMAN	50 kDa	3	1
Plasminogen activator inhibitor 1	PAI1_HUMAN	45 kDa	0	4
14-3-3 protein zeta/delta	1433Z_HUMAN	28 kDa	1	2
Rab GDP dissociation inhibitor alpha	GDIA_HUMAN	51 kDa	1	2
Keratin, type II cytoskeletal 1	K2C1_HUMAN	66 kDa	2	1
Laminin subunit alpha-3	LAMA3_HUMAN	37 kDa	0	3
14-3-3 protein epsilon	1433E_HUMAN	29 kDa	1	1
Fructose-bisphosphate aldolase C	ALDOC_HUMAN	39 kDa	1	1
Chromogranin-A	CMGA_HUMAN	51 kDa	1	1
Elongation factor 1-alpha 1	EF1A1_HUMAN	50 kDa	1	1
Filamin-A	FLNA_HUMAN	281 kDa	1	1
78 kDa glucose-regulated protein	GRP78_HUMAN	72 kDa	1	1
Hypoxanthine-guanine phosphoribosyltransferase	HPRT_HUMAN	25 kDa	1	1
Pyruvate kinase PKM	KPYM_HUMAN	58 kDa	1	1
Putative nucleoside diphosphate kinase	NDK8_HUMAN	16 kDa	1	1
Phosphatidylethanolamine-binding protein 1	PEBP1_HUMAN	21 kDa	1	1
Tropomyosin alpha-4 chain	TPM4_HUMAN	29 kDa	1	1
Complement C3	CO3_HUMAN	187 kDa	1	1
Creatine kinase B-type	KCRB_HUMAN	43 kDa	2	0
Clathrin heavy chain 1	CLH1_HUMAN	192 kDa	2	0
Fibrinogen alpha chain	FIBA_HUMAN	95 kDa	1	0
Protein strawberry notch homolog 2	SBNO2_HUMAN	150 kDa	1	0
ATP synthase subunit alpha, mitochondrial	ATPA_HUMAN	60 kDa	1	0
Flavin reductase (NADPH)	BLVRB_HUMAN	22 kDa	1	0
Dihydropyrimidinase-related protein 2	DPYL2_HUMAN	62 kDa	1	0
Rab GDP dissociation inhibitor beta	GDIB_HUMAN	51 kDa	1	0
Heterogeneous nuclear ribonucleoprotein K	HNRPK_HUMAN	51 kDa	1	0
Eukaryotic initiation factor 4A-I	IF4A1_HUMAN	46 kDa	1	0
NEDD8	NEDD8_HUMAN	9 kDa	1	0
POTE ankyrin domain family member E	POTEE_HUMAN	121 kDa	1	0
Peptidyl-prolyl cis-trans isomerase A	PPIA_HUMAN	18 kDa	1	0
Proteasome subunit alpha type-5	PSA5_HUMAN	26 Kda	1	0
Proteasome subunit alpha type-7-like	PSA7L_HUMAN	29 kDa	1	0
Ras-related protein Rab-11A	RB11A_HUMAN	24 kDa	1	0
Stathmin	STMN1_HUMAN	17 kDa	1	0
Tubulin beta-4A chain	TBB4A_HUMAN	50 kDa	1	0
T-complex protein 1 subunit theta	TCPQ_HUMAN	60 kDa	1	0
Transitional endoplasmic reticulum ATPase	TERA_HUMAN	89 kDa	1	0
Transketolase	TKT_HUMAN	68 kDa	1	0
14-3-3 protein theta	1433T_HUMAN	28 kDa	0	1
Cadherin-3	CADH3_HUMAN	91 kDa	0	1
Neutrophil gelatinase-associated lipocalin	NGAL_HUMAN	23 kDa	0	1
Nucleobindin-1	NUCB1_HUMAN	54 kDa	0	1
Phosphoglucomutase-1	PGM1_HUMAN	61 kDa	0	1
Peroxiredoxin-2	PRDX2_HUMAN	22 kDa	0	1
Tropomyosin alpha-3 chain	TPM3_HUMAN	33 kDa	0	1
Apolipoprotein A-I	APOA1_HUMAN	31 kDa	0	1
Transforming growth factor-beta-induced protein ig-h3	BGH3_HUMAN	75 kDa	0	1
Malate dehydrogenase, cytoplasmic	MDHC_HUMAN	36 kDa	1	0
Reticulocalbin-3	RCN3_HUMAN	37 kDa	1	0
Coagulation factor V	FA5_HUMAN	252 kDa	1	0
Transgelin	TAGL_HUMAN	23 kDa	1	0
Catenin alpha-2	CTNA2_HUMAN	105 kDa	0	1
PHD finger protein 7	PHF7_HUMAN	44 kDa	0	1
Protein OSCP1	OSCP1_HUMAN	45 kDa	1	0
