# Supplementary material for: Pathomimetic avatars reveal divergent roles of microenvironment in invasive transition of ductal carcinoma in situ
Source: Breast Cancer Res. 2017 May 15;19:56. doi: 10.1186/s13058-017-0847-0 (PMC5433063; doi:10.1186/s13058-017-0847-0)
Supplement: Supplementary file 13 — Proteomic analysis of conditioned media from 3D MEP and DCIS cultures. (PDF 57 kb) [file 13058_2017_847_MOESM13_ESM.pdf]

Additional File 13: Table S3. Proteomic Analysis of Conditioned Media from 3D MEP and DCIS Cultures.

Identified Proteins	UniProt Entry name	Molecular Weight (kDa)	3D Cultures				
			3D Media	MEPs	DCIS + MEPs	DCIS	Media
			# of peptides				
Vimentin	VIME_HUMAN	54	5	8	10	10	0
Hemoglobin subunit alpha	HBA_HUMAN	15	2	4	3	3	5
Serum albumin	ALBU_HUMAN	69	4	3	3	4	5
Heat shock cognate 71 kDa protein	HSP7C_HUMAN	71	1	3	5	5	1
Plasminogen activator inhibitor 1	PAI1_HUMAN	45	0	3	7	1	0
Laminin subunit beta-1	LAMB1_HUMAN	198	1	6	4	0	0
Transgelin	TAGL_HUMAN	23	0	4	3	3	1
Myosin-1	MYH1_HUMAN	22	1	3	6	3	0
Vasopressin-neurophysin 2-copeptin	NEU2_HUMAN	17	2	2	2	3	2
Actin, cytoplasmic 1	ACTB_HUMAN	42	0	1	2	4	0
Keratin, type I cytoskeletal 10	K1C10_HUMAN	59	6	0	2	0	0
14-3-3 protein epsilon	1433E_HUMAN	29	0	1	3	3	1
Phosphatidylethanolamine-binding protein 1	PEBP1_HUMAN	21	1	2	2	1	2
Hemoglobin subunit epsilon	HBE_HUMAN	16	1	2	1	2	1
Cystatin-C	CYTC_HUMAN	16	1	1	2	2	0
Triosephosphate isomerase	TPIS_HUMAN	31	0	1	2	2	2
Ubiquitin-60S ribosomal protein L40	RL40_HUMAN	15	0	1	1	1	0
Heat shock protein HSP 90-alpha	HS90A_HUMAN	85	0	1	2	1	0
Elongation factor 1-alpha 1	EF1A1_HUMAN	50	1	2	2	1	0
Secretogranin-2	SCG2_HUMAN	71	0	1	1	0	0
Antileukoproteinase	SLPI_HUMAN	14	1	0	2	2	0
Keratin, type II cytoskeletal 1	K2C1_HUMAN	66	4	1	1	0	0
ATP synthase subunit alpha, mitochondrial	ATPA_HUMAN	60	0	1	3	2	0
Calmodulin	CALM_HUMAN	17	0	0	1	2	1
Alpha-actinin-1	ACTN1_HUMAN	103	0	1	1	0	0
Peroxidasin homolog	PXDN_HUMAN	165	0	0	2	1	0
Peroxiredoxin-1	PRDX1_HUMAN	22	0	1	2	1	0
Metalloproteinase inhibitor 1	TIMP1_HUMAN	23	0	0	2	3	0
Transforming growth factor-beta-induced protein ig-h3	BGH3_HUMAN	75	0	0	2	1	0
Prothymosin alpha	PTMA_HUMAN	12	0	1	0	1	1
Nidogen-1	NID1_HUMAN	136	2	1	1	1	0
Protein DJ-1	PARK7_HUMAN	20	0	1	1	2	1
Tropomyosin beta chain	TPM2_HUMAN	33	1	1	2	1	0
40S ribosomal protein S3	RS3_HUMAN	27	0	1	2	2	0
Thrombospondin-1	TSP1_HUMAN	129	0	1	2	2	0
78 kDa glucose-regulated protein	GRP78_HUMAN	72	0	1	2	1	0
Basement membrane-specific heparan sulfate proteoglycan core protein	PGBM_HUMAN	469	0	1	1	1	0
Heterogeneous nuclear ribonucleoprotein K	HNRPK_HUMAN	51	0	1	1	1	0
SPARC	SPRC_HUMAN	35	0	1	1	1	0
Alpha-enolase	ENOA_HUMAN	47	0	1	2	1	0
Myosin regulatory light chain 12A	ML12A_HUMAN	20	0	0	1	2	0
Elafin	ELAF_HUMAN	12	0	0	2	1	0
Laminin subunit gamma-1	LAMC1_HUMAN	178	1	0	3	0	0
Lamin-B1	LMNB1_HUMAN	66	0	1	2	0	0
Myosin-9	MYH9_HUMAN	227	0	0	1	1	0
Protein FAM149B1	F149B_HUMAN	65	1	1	0	0	1
Transmembrane protein 79	TMM79_HUMAN	44	0	0	0	0	1
Macrophage migration inhibitory factor	MIF_HUMAN	12	0	0	1	1	0
Thioredoxin	THIO_HUMAN	12	0	0	1	1	0
Alpha-2-HS-glycoprotein	FETUA_HUMAN	39	0	2	1	0	0
Prelamin-A/C	LMNA_HUMAN	74	0	0	2	0	0
Ubiquitin carboxyl-terminal hydrolase isozyme L1	UCHL1_HUMAN	25	0	0	1	0	1
